# Identification of the prognostic value of Th1/Th2 ratio and a novel prognostic signature in basal-like breast cancer

**DOI:** 10.1186/s41065-023-00265-0

**Published:** 2023-01-25

**Authors:** Yu Xiao, Yi Huang, Jianping Jiang, Yan Chen, Changyuan Wei

**Affiliations:** 1 Affiliated Tumor Hospital, Guangxi Medical University, Nanning, Guangxi China; 2 Department of Thyroid and Breast Surgery, Shenzhen Second People’s Hospital, Shenzhen, Guangdong China; 3 Department of Research, Affiliated Tumor Hospital, Guangxi Medical University, Nanning, Guangxi China

**Keywords:** Breast cancer, Basal-like, Th1/Th2 balance, Bioinformatic analysis, Prognostic model

## Abstract

**Background:**

Breast cancer is a heterogeneous group of diseases. The polarization of CD4+ T helper (Th) lymphocytes (mainly Th1 and Th2) may differ in breast cancers with different outcomes, but this has not been fully validated.

**Methods:**

This study is a bioinformatic analysis, in which differentially expressed genes (DEGs) were identified in patients with low and high Th1/Th2 ratios. And then, DEG functions, hub genes and independent predictors were determined.

**Results:**

Low Th1/Th2 ratio was associated with poor outcome in Luminal A and basal-like breast cancer (*p* < 0.05). GSEA and KEGG analysis of DEGs obtained from comparing low and high Th1/Th2 ratios illuminated downregulation of immune-related gene sets and pathways affecting Th1/Th2 balance toward Th2 polarization (*p* < 0.05). Survival and Cox analyses of all the DEGs confirmed CCL1 and MYH6 were independent protective factors and IFNK and SOAT2 were independent risk factors for basal-like breast cancer (95%CI: 1.06–2.5, *p* = 0.026). Then a four-gene signature was constructed and achieved a promising prognostic value (C-index = 0.82; AUC = 0.826).

**Conclusions:**

Low Th1/Th2 ratio predicts poor outcome in Luminal A and Basal-like breast cancer, and downregulation of immune-related gene sets and pathways contribute to Th1/Th2 balance toward Th2 polarization. CCL1, MYH6, IFNK, and SOAT2 have an independent prognostic value of survival outcome and might be novel markers in basal-like breast cancer.

**Supplementary Information:**

The online version contains supplementary material available at 10.1186/s41065-023-00265-0 .

## Introduction

 Breast cancer is one of the most frequently diagnosed cancers worldwide and has become a global health concern for women [ 1 ]. Through continuous scientific efforts, a foundation for the treatment of breast cancer has been laid, mainly consisting of surgery, chemotherapy, endocrine therapy, and radical therapy [ 2 ]. Although a relatively better outcome has been achieved with breast cancer compared with other solid tumors, and a 5-year survival rate of over 80% is a remarkable success, there are still patients with poor prognosis [ 3 ],while immune related gene signature may contribute to a better prognostic assessment. 

 Immune-related studies are widely used in oncology, among which the balance of T helper (Th)1/Th2 lymphocytes has been investigated intensively and found to be linked with other conditions such as inflammation, immune diseases, and tumors [ 4, 5 ]. Previous studies have reported that Th1 cells produce interleukin (IL)-2, tumor necrosis factor (TNF)-β, and interferon (IFN)-γ and activate cytotoxic T lymphocytes (Tc), NK cells, macrophages, and monocytes, playing an important role in the immune response against tumors. Th2 cells produce IL-4, IL-5, IL-6, IL-9, IL-10, and IL-13 and act against Th1 cells [ 6 ]. A shift of Th1/Th2 cell subsets toward Th2 cells in malignant tumors has been reported [ 7, 8 ]. Furthermore, T cell differentiation is a complex process stimulated by different antigens, cytokines, or antigen-presenting cells. Th0 cells can be transformed into Th1 or Th2 cells, or promote Th1 cell transformation to Th2 cells, thus, causing a shift in Th1/Th2 balance. Among the cytokines associated with the Th1/Th2 balance, IL-4 and IFN-γ play a key role in the differentiation of Th0 cells into Th1 and Th2. When the IL-4 level is high, Th0 cells mainly differentiate into Th2 cells. While IL-4 is deficient, the expression of IFN-γ increases and induces differentiation into Th1 cells. IFN-γ secreted by Th1 and IL-4 and IL-10 secreted by Th2 can not only promote their own differentiation and maturation but can also inhibit the differentiation and maturation of each other and form a regulatory network with other factors [ 9, 10 ]. 

 Emerging evidence has confirmed the predictive value in the prognostic and drug efficacy of Th1/Th2 balance in breast cancer [ 11, 12 ]. However, the differences of gene expression pattern at different levels of the Th1/Th2 ratio and the mechanism behind them are still not fully clarified. This study aims to investigate the prognostic value of the Th1/Th2 ratio in different breast cancer subtypes and further explore the prognostic value of Th1/Th2 balance related gene signature in breast cancer. 

## Materials and methods

### Data source

 Breast cancer (BRCA) RNA-seq data were downloaded from TCGA ( http://portal.gdc.cancer.gov/ , v31.0) and clinical data were downloaded from the TCGA Pan-Cancer Clinical Data Resource (TCGA-CDR) [ 13 ]. 

 The corresponding abundance data of Th1 and Th2 cells was downloaded from ImmuCellAI ( http://bioinfo.life.hust.edu.cn/ImmuCellAI) [14 ]. 

 Independent dataset GSE202203 of basal-like breast cancer were downloaded from the Gene Expression Omnibus (GEO) ( https://www.ncbi.nlm.nih.gov/geo/ ). 

### Data preparing and survival analysis

 The data of 1075 patients who were female and had complete overall survival (OS) information were acquired from the TCGA BRCA dataset. The RNA-seq counts data underwent a normalization procedure using variance stabilizing transformation in R and was annotated by gencode.v22.annotation downloaded from https://gdc.cancer.gov/ . According to the PAM50 criteria, patients were classified into luminal A (LumA), luminal B (LumB), Her2 overexpressed (Her2), basal-like (Basal), and normal-like (Normal) subtypes [ 15 ]. The Th1/Th2 ratio was calculated and survival analyses comparisons with different levels of Th1/Th2 ratio were performed in different breast cancer subtypes (clinical details in Supplementary data Table s 1 ). 

### Identification of DEGs

 Setting criteria as *p* value < 0.05 and |Log2FC| > 1.5, DEGs were identified by the R package “DEseq2” comparing the low Th1/Th2 ratio group with the high Th1/Th2 ratio group, and genes with an average count value lower than 1 were excluded. The DEGs were visualized as a heatmap and MAplot using “pheatmap”, “ggplot2”, and “ggrepel” packages in R [ 16 ]. 

### GSEA and KEGG enrichment analyses

 Gene set enrichment analysis (GSEA) and analysis using Kyoto Encyclopedia of Genes and Genomes (KEGG) of whole DEGs were conducted to annotate gene functions [ 17 ]. All GSEA presented in this study were based on hallmark gene sets using the R package “clusterProfiler” [ 18 ]. Both adjusted *P* value and FDR value < 0.05 were considered as indicating significant enrichment. DEGs meeting the criteria of *p* value < 0.05 and |Log2FC| > 1.5 were analyzed for KEGG enrichment and the enriched pathways were visualized by the R package “clusterProfiler” and “pathview” [ 19 ]. Both an adjusted *P* value and FDR value of < 0.05 were considered as indicating a significant enrichment. 

### Risk score signature construction and validation

Univariate cox analysis and survival analysis by Log-rank test were used to filter potential predictive markers and multivariate cox regression analysis was used in risk model construction. Then the predictive ability was assessed by receiver operating characteristic curve (ROC) to compare Th1/Th2 ratio, tumor stage, and age. Data in this study were randomly divided into a training set and testing set for risk model construction and validation. The risk score signature was assessed by the following formula:

$$\mathrm{Risk}\;\mathrm{score} = {\sum}_{i=1}^n Coefi\ast \mathit{\exp}(i)$$

where *Coefi* is the multivariate Cox regression coefficient for the target mRNA and *exp*(*i*) is the expression value of each mRNA. According to the risk score signature cutoff point calculated by the “ROC” method in the R package “ggrisk” in the training set, all patient samples were divided into high-risk and low-risk groups. 

The risk score signature was validated in testing set and the entire set, and then was further validated in an independent dataset GSE202203 with 288 cases basal-like breast cancer from GEO.

### Statistical analyses

Survival curves were generated using the Kaplan-Meier method with R package “survival”. The best cut points of variates of survival analysis were evaluated by the R package “survminer”. The heatmap was performed by the R package “pheatmap” and the MA plot was constructed by “ggplot2” and “ggrepel”. The t test, chi-square test, and Fisher’s Exact test were used in the variance analyses. Cox analysis was used for multivariate analysis and correlation analysis was performed by the Spearman method. A risk score plot was constructed by using the R package “ggrisk” for Cox regression. All the statistical analyses were performed by R (version 4.1.0) and two-tailed *P* < 0.05 was considered as the standard for statistical significance.

## Results

### Abundance of Th1 and Th2 cells and survival analysis

**Fig. 1:**
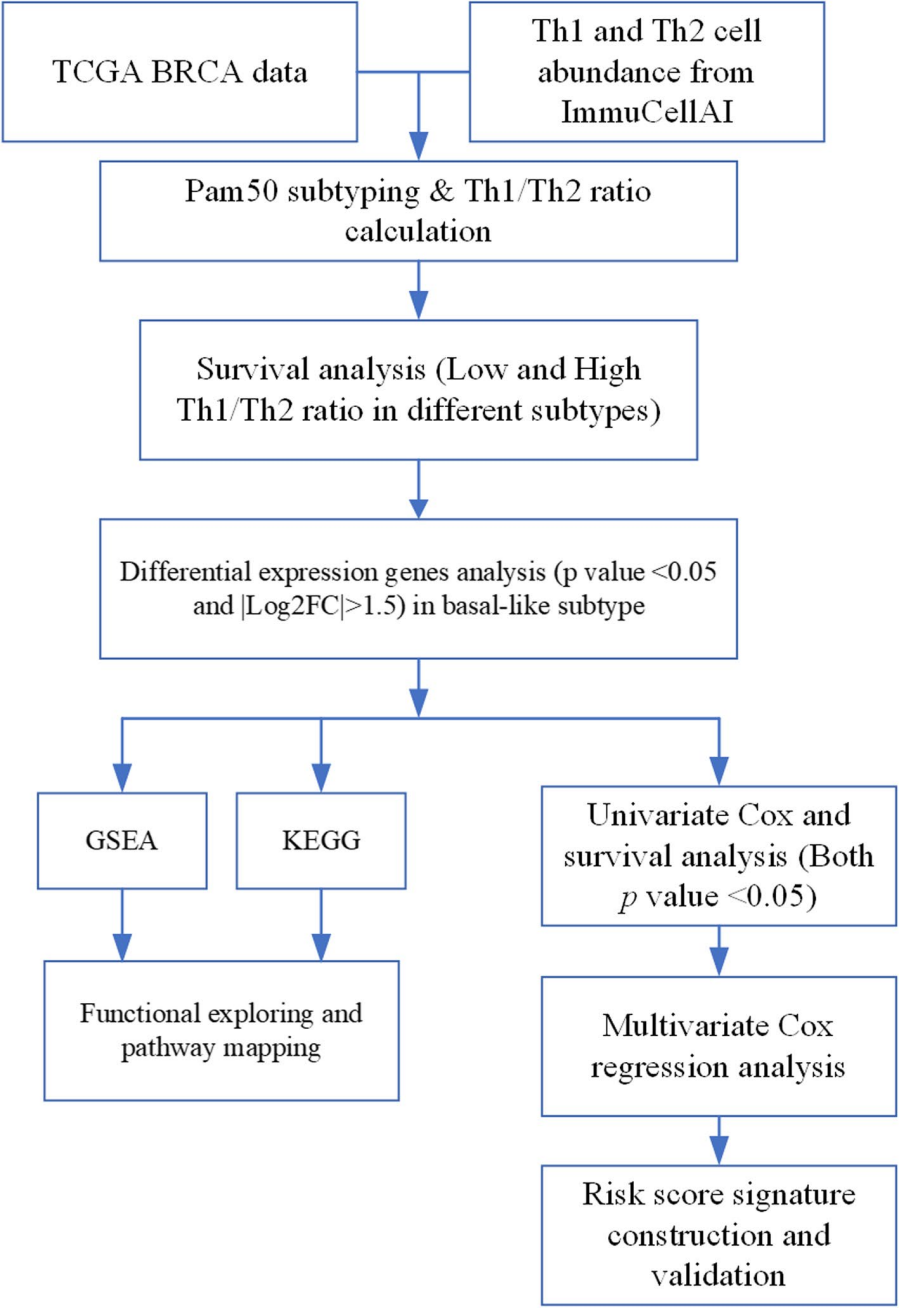
Flow chart of this study

**Fig. 2:**
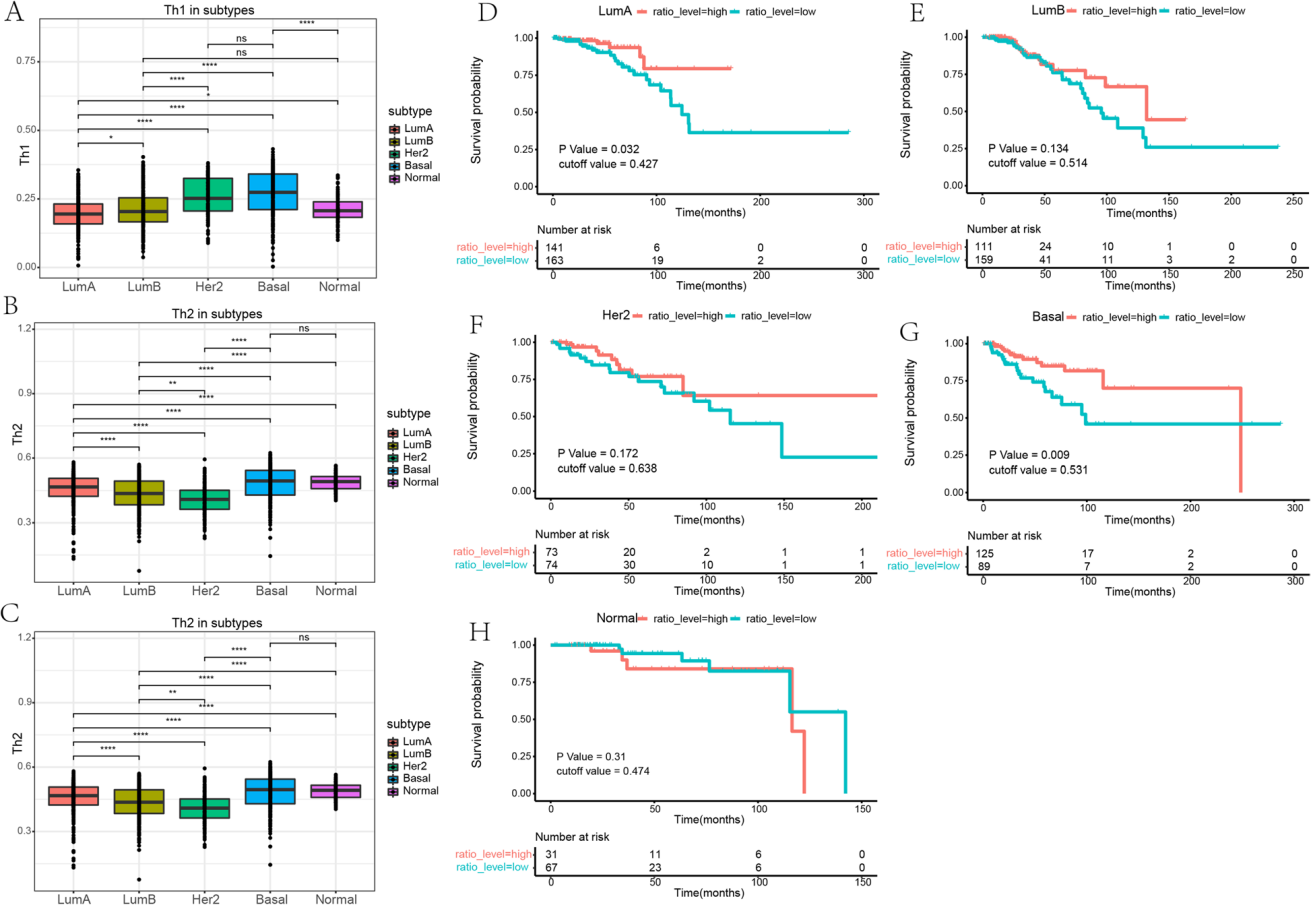
Evaluation of Th1 and Th2 cell abundance and the prognostic value of Th1/Th2 ratio in different subtypes of breast cancer. **a**, **b** Abundance of Th1 and Th2 cells in different subtypes of breast cancer. **c** Th1/Th2 ratio level in different subtypes of breast cancer. **d**–**h** The survival analyses for high and low Th1/Th2 ratio in different subtypes of breast cancer. * p < 0.05; ** p < 0.01; *** p < 0.001; **** p < 0.0001

 The detailed workflow of this study is shown in Fig.  1 . The abundance of Th1 and Th2 cells and the Th1/Th2 ratio level were analyzed with TCGA BRCA data (Fig.  2 A–C). Grouped by high and low Th1/Th2 ratio, survival analyses were performed and showed that a low Th1/Th2 ratio was a poor prognostic factor in LumA and Basal subtypes (*P* < 0.05), whereas the prognostic value was not significant in LumB, Her2, and Normal subtypes (excluding patients with an OS time < 30 days) (Fig. 2 D–H). We identified that the cutoff value of the Th1/Th2 ratio was 0.531 for the Basal subtype and extracted the Basal subtype breast cancer data for further analyses (clinical details of Basal subtype data in Table  1 ).

**Table 1:** Clinical details

	High Th1/Th2 ratio	Low Th1/Th2 ratio	*p*
n	125	89	
Age (median [IQR]) *	55.00 [48.00, 64.00]	51.00 [46.00, 62.00]	0.105
Stage (%) *			
Stage I/II	106 (84.8)	64 (71.9)	0.365
Stage III/IV	18 (14.4)	20 (22.5)	
Stage X	1 (0.8)	5 (5.6)	
Histological type (%) *			
Infiltrating Ductal Carcinoma	106 (84.8)	74 (83.1)	0.398
Infiltrating Lobular Carcinoma	7 (5.6)	0 (0.0)	
Other	12 (9.6)	15 (16.9)	
Menopause status (%)			
Pre	39 (31.2)	30 (33.7)	0.535
Post	79 (63.2)	51 (57.3)	
Unclear	7 (5.6)	8 (9.0)	

* Data of age didn’t fit the normal distribution, and the *p* value was calculated by non-norm method; *p* value of data of stage and histological type were calculated by Fisher’s Exact Test

### DEG identification

**Fig. 3:**
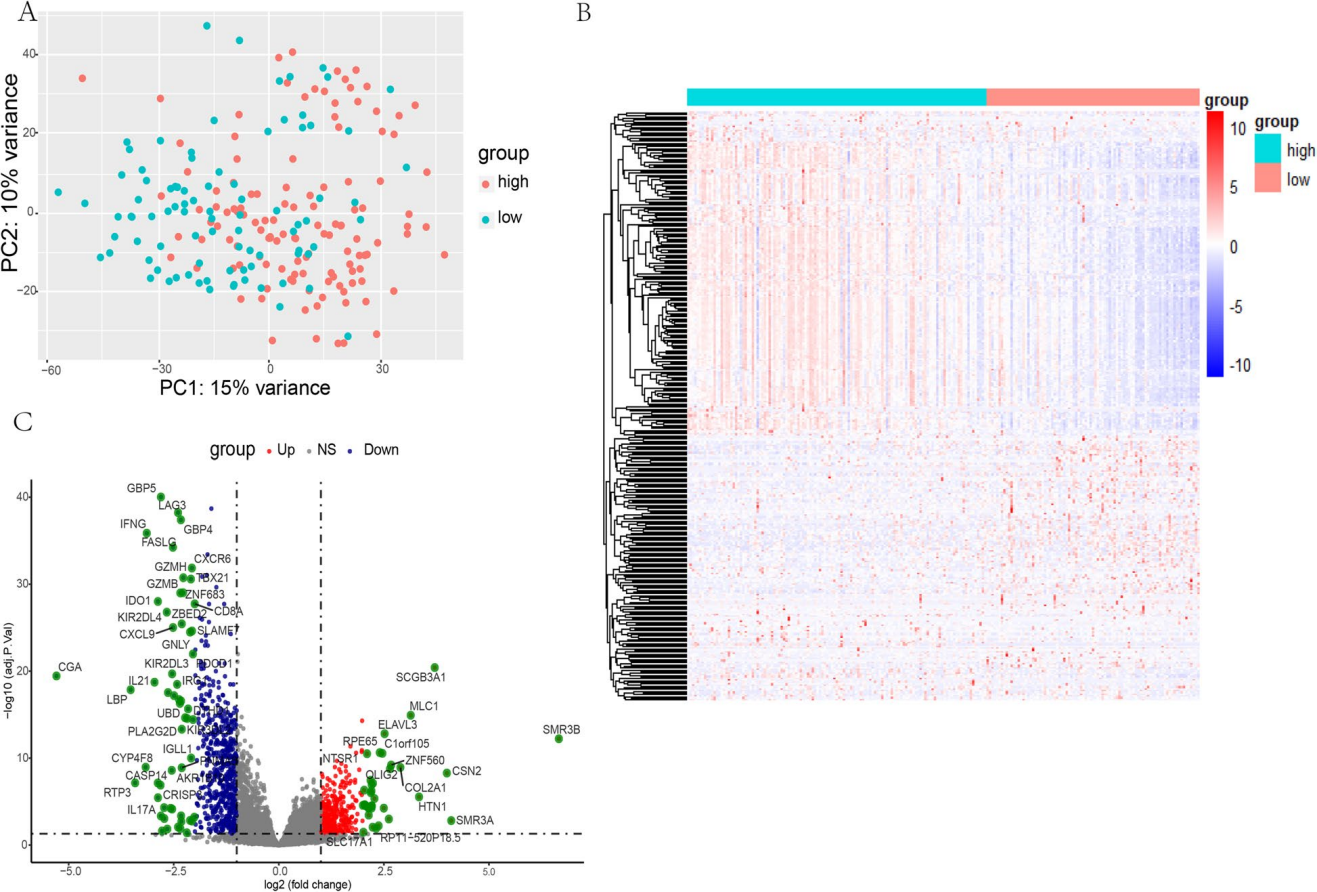
Identification of differentially expressed genes (DEGs). **a** PCA shows a 15% variance between groups of low and high Th1/Th2 ratio. **b**-**c** Heatmap, volcano plot exhibit expression status and distribution of DEGs that meet the criteria of *p* value < 0.05 and |Log2FC| > 1.5

 A total of 332 DEGs were identified from 19,495 protein-coding genes in the Basal subtype from the low Th1/Th2 ratio group in comparison with the high Th1/Th2 ratio group (DEG details in Supplementary data Table S 2 ). A heatmap of the DEGs was constructed and then volcano plot was conducted to reveal the significance of DEGs (Fig.  3 ).

### GSEA and KEGG analyses

**Fig. 4:**
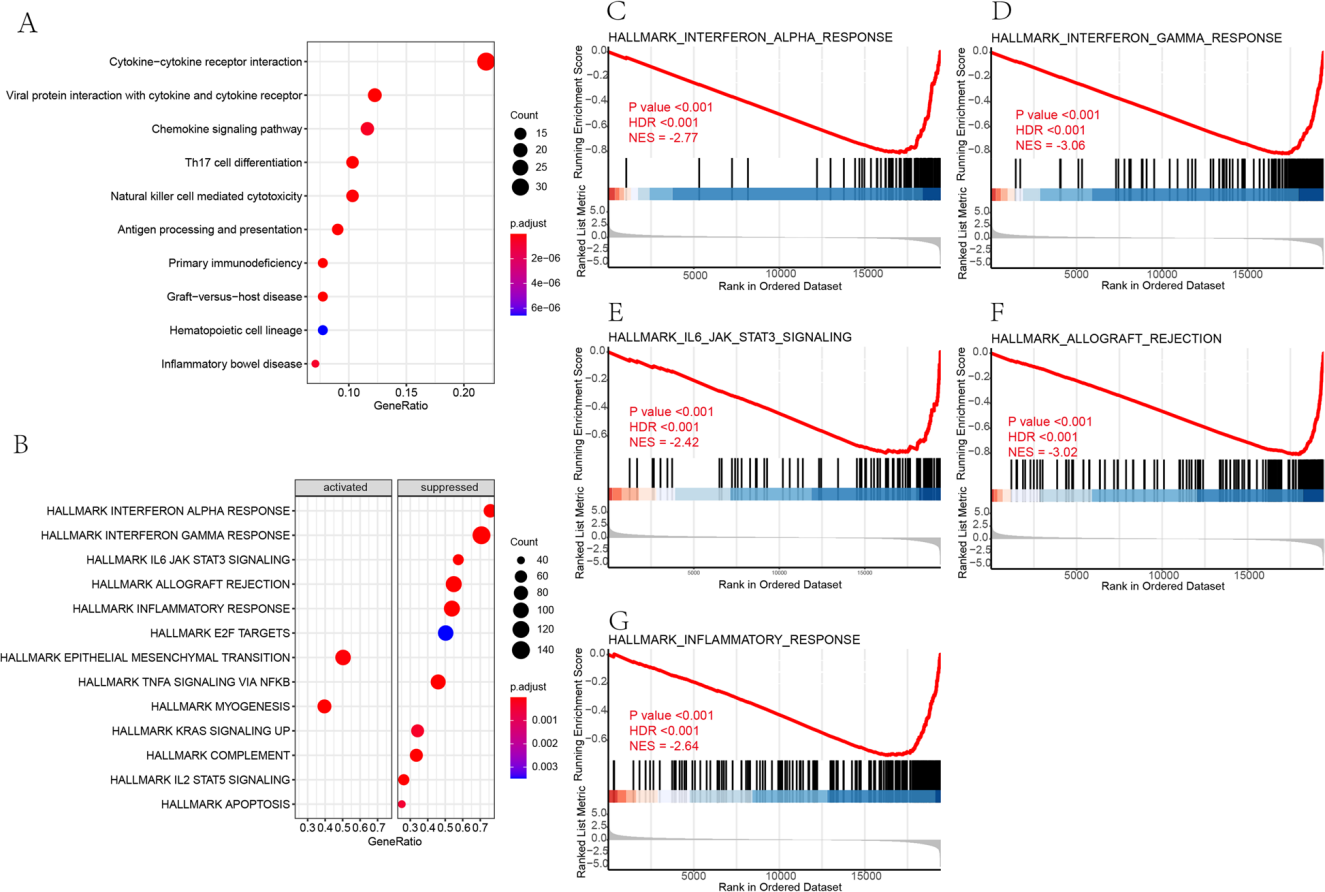
Gene set enrichment analysis (GSEA) and KEGG analysis. **a** Top 10 enriched pathways in KEGG analysis measured by gene ratio. **b** Enriched hallmark gene sets with FDR < 0.05. **c**-**g** Top five hallmark gene sets enriched in GSEA

 To clarify the function and related pathways of these DEGs, we conducted GSEA and KEGG analysis. Enrichments were detected in the downregulation of the IFN-γ response, allograft rejection, inflammatory response, IFN-α response, IL6 JAK STAT3 signaling, complement, TNFα signaling via NF-κB, IL2 STAT5 signaling, apoptosis, KRAS signaling, and E2F target gene sets, and in the upregulation of myogenesis and epithelial-mesenchymal transition by GSEA (Table  2 & Fig.  4 B). Top five gene sets were shown in Fig. 4 C-G. In the KEGG analysis of 332 DEGs, 22 pathways were enriched and the top 10 enriched pathways estimated by gene ratio were cytokine-cytokine receptor interaction, viral protein interaction with cytokine and cytokine receptor, primary immunodeficiency, graft versus host disease, Th17 cell differentiation, antigen processing and presentation, natural killer cell-mediated cytotoxicity, chemokine signaling pathway, inflammatory bowel disease, and hematopoietic cell lineage (Table  3 & Fig. 4 A).

**Table 2:** GSEA results

ID	Set Size	Enrichment Score	NES	p.adjust	FDR	rank	Leading edge
INTERFERON_GAMMA_RESPONSE	198	− 0.819	−3.060	< 0.001	< 0.001	2420	tags = 71%, list = 12%, signal = 63%
ALLOGRAFT_REJECTION	195	−0.811	−3.020	< 0.001	< 0.001	1459	tags = 55%, list = 8%, signal = 51%
INFLAMMATORY_RESPONSE	197	−0.706	−2.640	< 0.001	< 0.001	3096	tags = 54%, list = 16%, signal = 46%
INTERFERON_ALPHA_RESPONSE	95	−0.813	−2.766	< 0.001	< 0.001	2409	tags = 76%, list = 12%, signal = 67%
IL6_JAK_STAT3_SIGNALING	87	−0.723	−2.422	< 0.001	< 0.001	3212	tags = 57%, list = 17%, signal = 48%
COMPLEMENT	200	−0.588	−2.199	< 0.001	< 0.001	2147	tags = 34%, list = 11%, signal = 30%
TNFA_SIGNALING_VIA_NFKB	198	−0.586	−2.188	< 0.001	< 0.001	3628	tags = 46%, list = 19%, signal = 38%
IL2_STAT5_SIGNALING	194	−0.537	−2.000	< 0.001	< 0.001	2205	tags = 26%, list = 11%, signal = 24%
MYOGENESIS	197	0.483	1.888	< 0.001	< 0.001	3889	tags = 40%, list = 20%, signal = 32%
EPITHELIAL_MESENCHYMAL_TRANSITION	197	0.445	1.737	< 0.001	< 0.001	4246	tags = 50%, list = 22%, signal = 40%
KRAS_SIGNALING_UP	193	−0.454	− 1.694	< 0.001	< 0.001	3002	tags = 34%, list = 15%, signal = 29%
APOPTOSIS	159	−0.467	− 1.701	< 0.001	< 0.001	3088	tags = 25%, list = 16%, signal = 21%
E2F_TARGETS	195	−0.417	−1.555	0.004	0.003	6328	tags = 50%, list = 33%, signal = 34%
GLYCOLYSIS	197	0.347	1.355	0.041	0.027	4328	tags = 25%, list = 22%, signal = 20%

**Table 3:** KEGG analysis result of 22 enriched pathways

ID	Description	Gene Ratio	Bg Ratio	p.adjust	FDR	Rich Factor ^a^
hsa04060	Cytokine-cytokine receptor interaction	34/155	295/8108	< 0.001	< 0.001	0.115
hsa04061	Viral protein interaction with cytokine and cytokine receptor	19/155	100/8108	< 0.001	< 0.001	0.190
hsa05340	Primary immunodeficiency	12/155	38/8108	< 0.001	< 0.001	0.316
hsa05332	Graft-versus-host disease	12/155	42/8108	< 0.001	< 0.001	0.286
hsa04659	Th17 cell differentiation	16/155	107/8108	< 0.001	< 0.001	0.149
hsa04612	Antigen processing and presentation	14/155	78/8108	< 0.001	< 0.001	0.179
hsa04650	Natural killer cell mediated cytotoxicity	16/155	131/8108	< 0.001	< 0.001	0.122
hsa04062	Chemokine signaling pathway	18/155	192/8108	< 0.001	< 0.001	0.094
hsa05321	Inflammatory bowel disease	11/155	65/8108	< 0.001	< 0.001	0.169
hsa04640	Hematopoietic cell lineage	12/155	99/8108	< 0.001	< 0.001	0.121
hsa04658	Th1 and Th2 cell differentiation	11/155	92/8108	< 0.001	< 0.001	0.119
hsa04514	Cell adhesion molecules	13/155	149/8108	< 0.001	< 0.001	0.087
hsa04660	T cell receptor signaling pathway	10/155	104/8108	< 0.001	< 0.001	0.096
hsa05235	PD-L1 expression and PD-1 checkpoint pathway in cancer	9/155	89/8108	< 0.001	< 0.001	0.101
hsa05320	Autoimmune thyroid disease	7/155	53/8108	< 0.001	< 0.001	0.132
hsa05330	Allograft rejection	6/155	38/8108	< 0.001	< 0.001	0.158
hsa04940	Type I diabetes mellitus	6/155	43/8108	0.002	0.001	0.139
hsa05323	Rheumatoid arthritis	8/155	93/8108	0.004	0.003	0.086
hsa05143	African trypanosomiasis	5/155	37/8108	0.006	0.006	0.135
hsa04672	Intestinal immune network for IgA production	5/155	49/8108	0.022	0.019	0.102
hsa04630	JAK-STAT signaling pathway	9/155	162/8108	0.034	0.030	0.056
hsa05416	Viral myocarditis	5/155	60/8108	0.049	0.043	0.083

^a^ Rich Factor = $$\frac{counts\ of\ DEGs\ enriched\ in\ one\ pathway}{counts\ of all\ the\ genes\ related\ in\ one\ pathway}$$

 We discovered that most of the enriched pathways were immune-related. A low Th1/Th2 ratio was associated with the downregulation of nearly all the enriched pathways mentioned above. In particular, the enrichment of the Th1 and Th2 cell differentiation pathway showed that the DEGs identified in this study were associated mostly with the downregulation of Th1 cell differentiation, which leads to Th1/Th2 polarization toward Th2 cell (Supplementary data figure s 9 ). 

 Details of pathways enriched are shown in Supplementary data Figure S 1 -S 22 , other hallmark gene sets enriched are shown in Supplementary data Figure S 23 and the information of specific genes related to each gene set and pathway are in Supplementary data Tables S 3 and S 4 . 

### Cox and survival analysis of DEGs

**Fig. 5:**
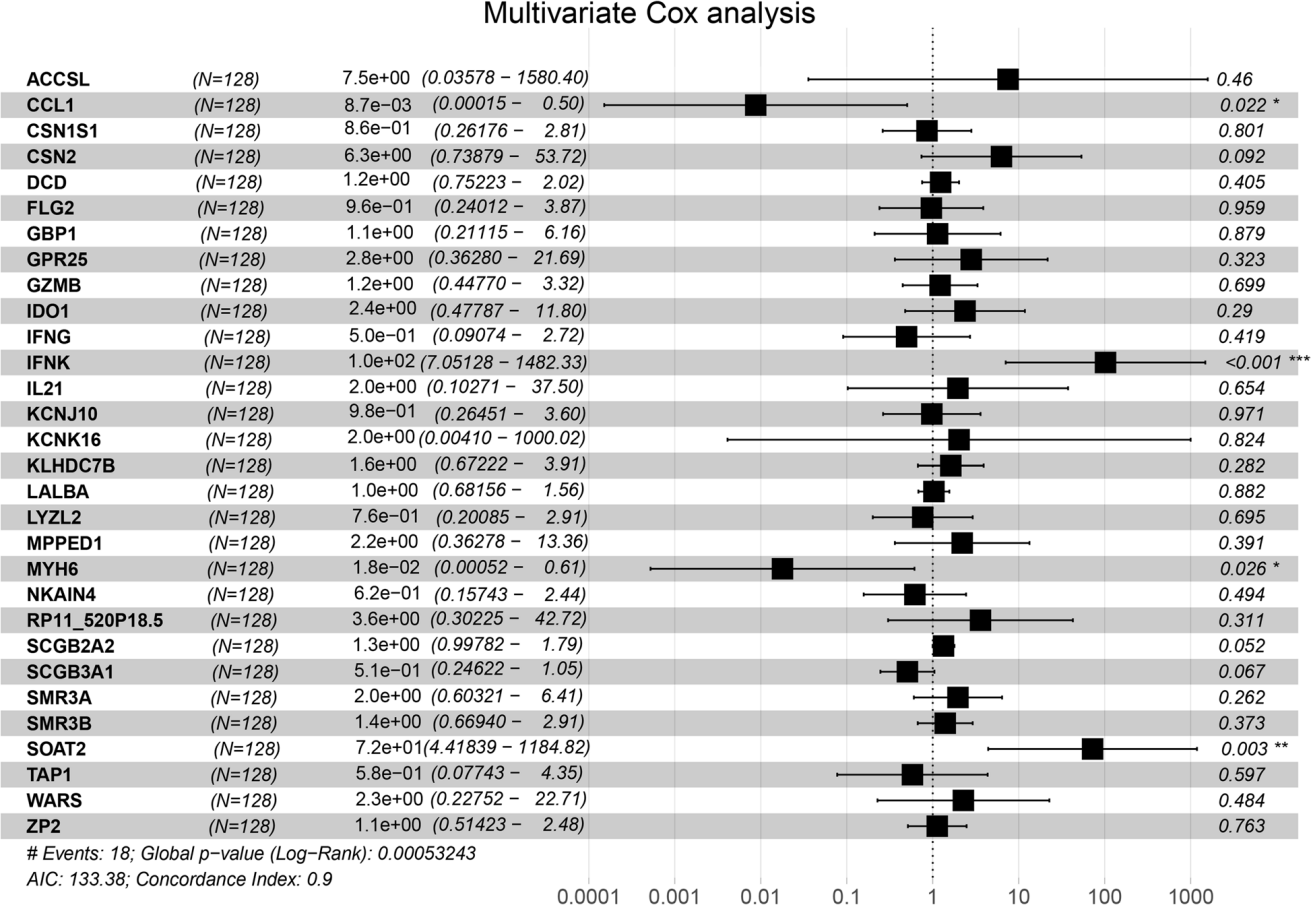
Multivariate Cox analysis of 30 genes. Multivariate Cox analysis confirmed that CCL1, MYH6 are independent protective factors and IFNK, SOAT2 is independent risk factors for basal-like breast cancer. * p < 0.05; ** p < 0.01; *** p < 0.001

**Fig. 6:**
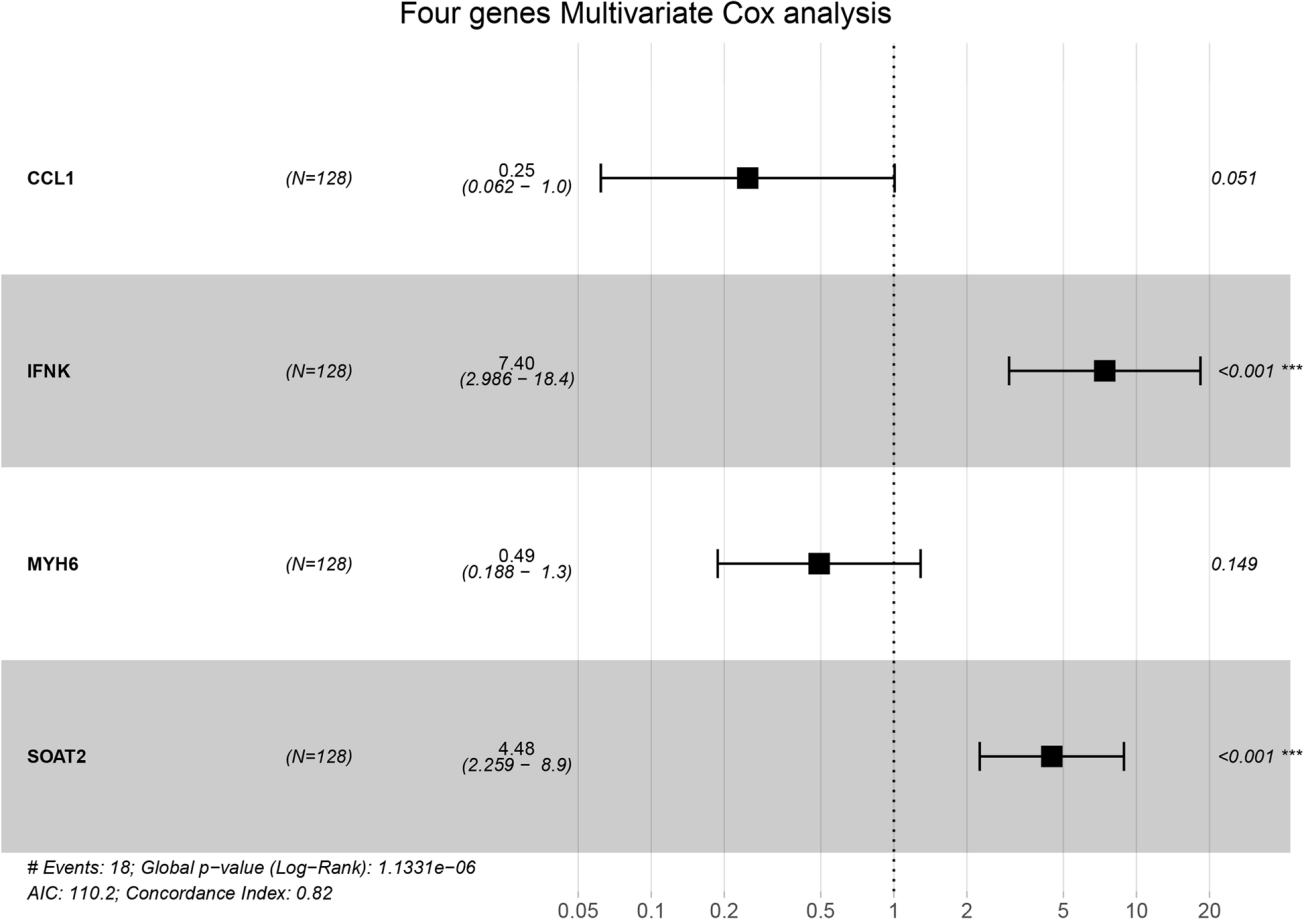
COX progression analysis for CCL1, IFNK, MYH6 and SOAT2. Constructing a four-gene signature by the four genes including CCL1, IFNK, MYH6 and SOAT2. * p < 0.05; ** p < 0.01; *** p < 0.001

**Fig. 7:**
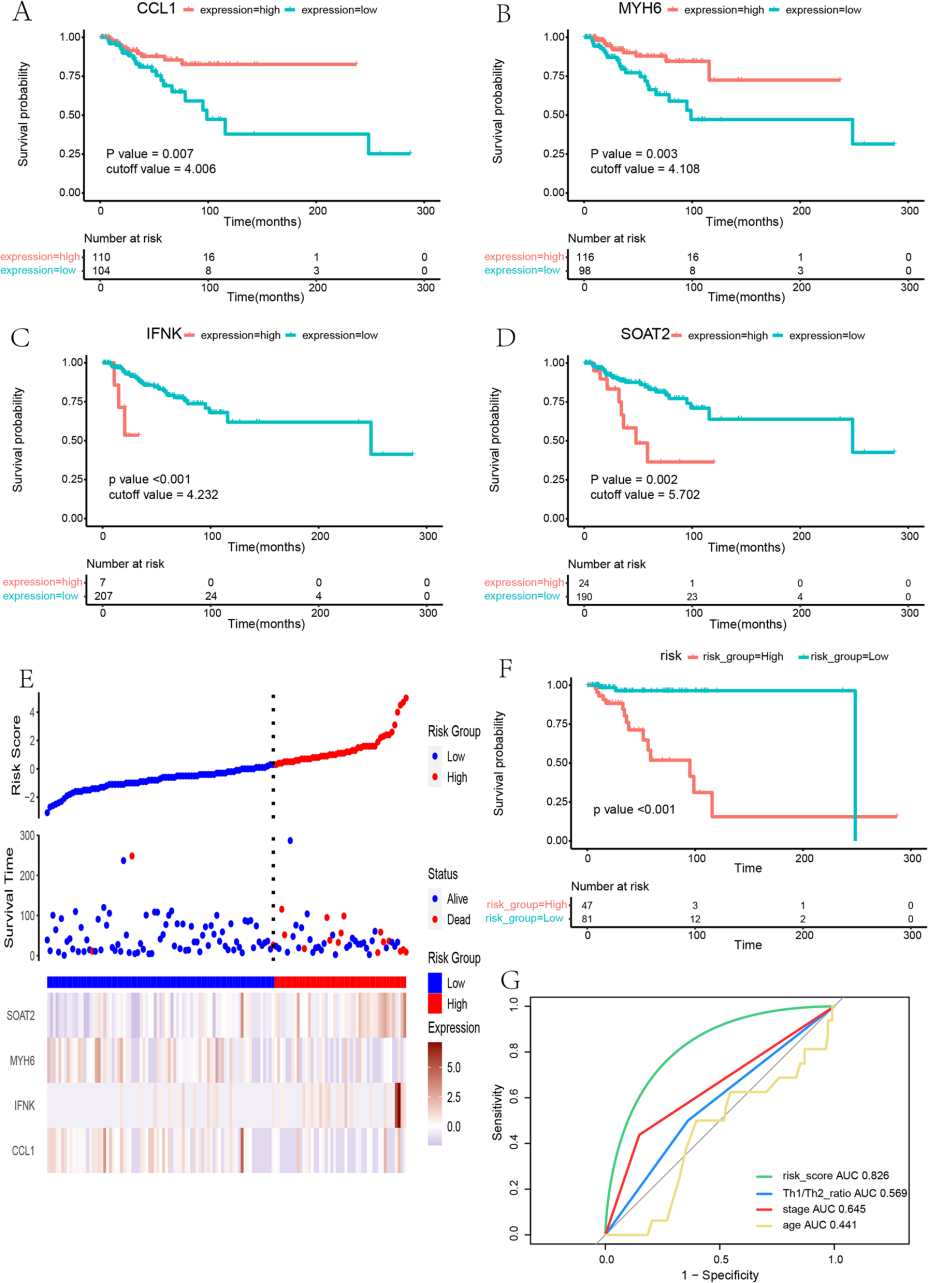
**a**-**d** Survival analysis showed that low expression of CCL1, MYH6 and high expression of IFNK, SOAT2 indicated a poor outcome of basal-like breast cancer. **e**-**g** Model construction in training set: Distribution of the risk score and patterns of survival status, survival time and expression of the 4 genes between the high and low risk groups; Survival curve of OS for High and low risk groups; ROC for comparison between risk model and Th1/Th2 ratio, tumor stage and age

 Univariate Cox analysis and survival analysis by Log-rank test were performed with the DEGs. 30 genes with a *p* value < 0.05 in both univariate Cox analysis and survival analysis were identified (Table  4 ). Then, multivariate Cox regression analysis was used to determine the independent predictive values of the 30 genes in survival outcomes (Fig.  5 ). The data were randomly divided into a training set and testing set in a ratio of 6:4 (training set: 128 patients; testing set: 86 patients). We confirmed that, for basal subtype breast cancer, CCL1 (95%CI: 0.00–0.50, *p* = 0.022) and MYH6 (95%CI: 0.00–0.61, *p* = 0.026) were independent protective factors while IFNK (95%CI: 7.05–1482.33, *p* < 0.001) and SOAT2 (95%CI: 4.42–1184.82, *p* = 0.003) were an independent risk factor in a training set with Cox regression analysis and survival analyses with the four genes mentioned above (Fig.  6 & 7 A-D).

**Table 4:** Genes both significant in univariate cox analysis and survival analysis

Gene name	HR	95% CI	*p*value_cox	*p*value_survival
ACCSL	2.7	(1.4–5)	0.003	< 0.001
CCL1	0.37	(0.17–0.83)	0.016	0.007
CSN1S1	1.1	(1–1.3)	0.033	< 0.001
CSN2	1.1	(1–1.3)	0.043	0.001
DCD	1.2	(1–1.3)	0.015	0.005
FLG2	1.4	(1.1–1.9)	0.013	0.001
GBP1	0.74	(0.59–0.91)	0.006	< 0.001
GPR25	0.56	(0.34–0.92)	0.021	0.011
GZMB	0.81	(0.68–0.96)	0.018	0.006
IDO1	0.86	(0.75–1)	0.049	0.006
IFNG	0.75	(0.58–0.98)	0.034	0.005
IFNK	1.5	(1.1–2.1)	0.014	< 0.001
IL21	0.46	(0.24–0.87)	0.018	0.003
KCNJ10	0.72	(0.55–0.94)	0.015	0.002
KCNK16	5	(2.3–11)	< 0.001	< 0.001
KLHDC7B	0.83	(0.72–0.96)	0.012	< 0.001
LALBA	1.1	(1–1.2)	0.024	0.004
LYZL2	1.6	(1.3–2.1)	< 0.001	< 0.001
MPPED1	1.3	(1–1.7)	0.037	< 0.001
MYH6	0.4	(0.21–0.77)	0.006	0.003
NKAIN4	1.3	(1–1.7)	0.048	< 0.001
RP11_520P18.5	1.9	(1.2–2.9)	0.003	0.007
SCGB2A2	1.1	(1–1.2)	0.026	0.001
SCGB3A1	1.2	(1–1.3)	0.028	0.001
SMR3A	1.2	(1–1.5)	0.042	0.005
SMR3B	1.2	(1.1–1.3)	< 0.001	< 0.001
SOAT2	1.4	(1.1–1.9)	0.020	0.002
TAP1	0.78	(0.62–1)	0.046	0.004
WARS	0.68	(0.49–0.94)	0.021	0.008
ZP2	1.2	(1–1.5)	0.043	0.003

### Risk score signature construction and validation

A Cox proportional-hazards model (a four-gene signature) comprising CCL1, IFNK, MYH6, and SOAT2 was constructed in a training set with the formula:

$$\mathrm{Risk}\;\mathrm{score}=\;-1.3870\:\times\:\exp(\mathrm{CCL}1)\:+\:2.0019\:\times\:\exp(\mathrm{IFNK})\:-\:0.7089\:\times\:\:\exp\;\mathrm{MYH}6)\:+\:1.4993\:\times\:\exp(\mathrm{SOAT}2)$$

**Fig. 8:**
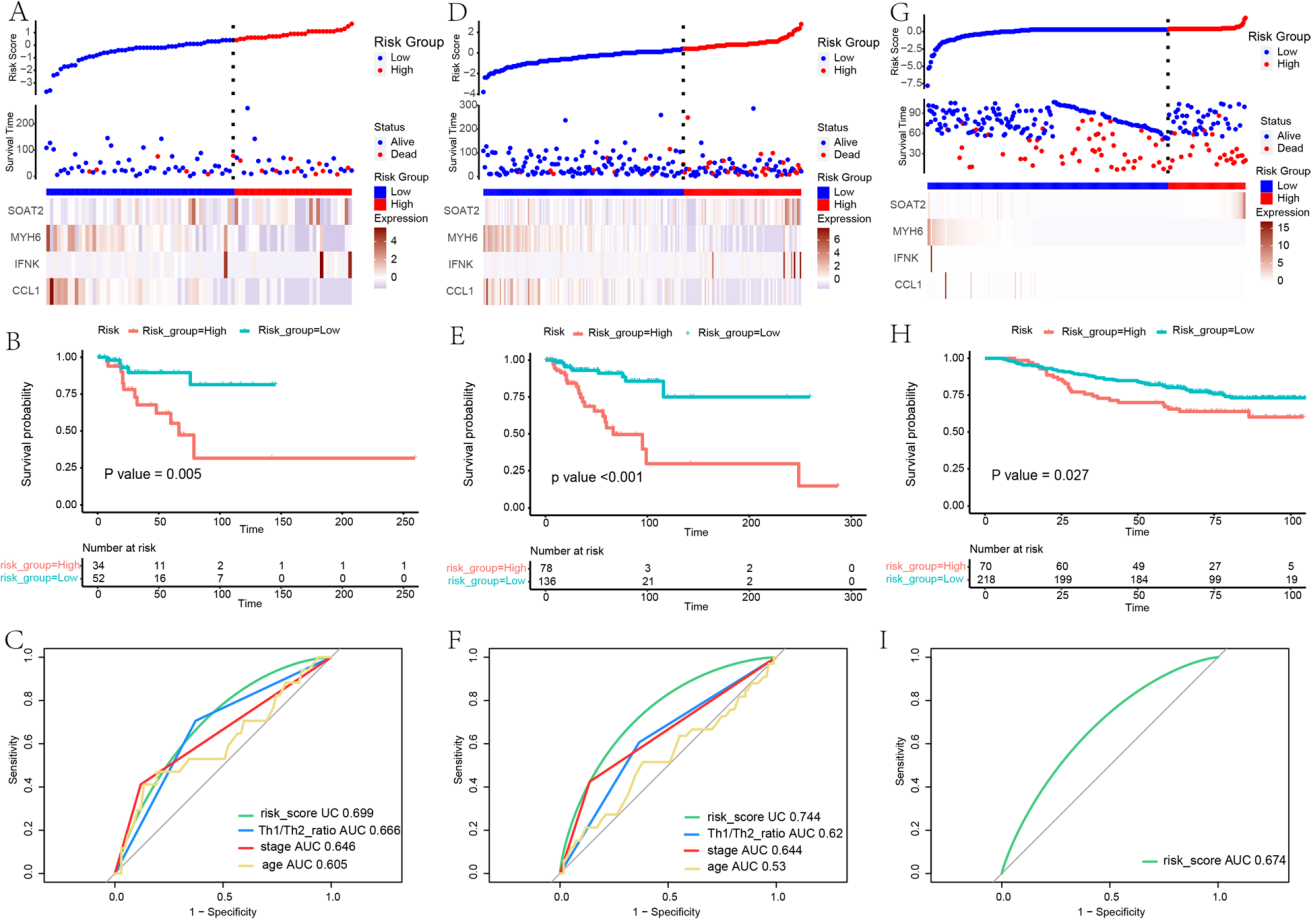
Internal and external validation. **a**-**c** Validation in testing set. **d**-**f** Validation in entire set. **g**-**i** Validation in an independent dataset GSE202203 from GEO

By calculating the risk score, patients were regrouped into high-risk and low-risk training sets, and this model achieved a concordance index (C-index) of 0.82. A risk score plot showed the distribution of patients (Fig.  7 E). The survival analysis showed significant differences between the high and low-risk groups (*p* < 0.001) (Fig. 7 F). ROC analysis showed a superior predictive ability when comparing the four-gene signature with the Th1/Th2 ratio, tumor stage, and age (ROC of risk score: 0.826) (Fig. 7 G). Then model validation was conducted in the testing set, the entire set. Similar results of the risk score possessing maximum area under the curve were achieved (ROC of the four-gene signature in testing set: 0.699; ROC of the four-gene signature in entire set: 0.744) (Fig.  8 A-F). ROC comparison in a different data set is listed in Table  5 .

**Table 5:** ROC comparison between Risk Score and other factors

Training set			Testing set			Entire set		
ROC1	ROC2	P	ROC1	ROC2	P	ROC1	ROC2	P
Risk Score	Th1/Th2 Ratio	< 0.05	Risk Score	Th1/Th2 Ratio	< 0.05	Risk Score	Th1/Th2 Ratio	< 0.05
	Tumor Stage	< 0.05		Tumor Stage	0.667		Tumor Stage	0.154
	Age	< 0.05		Age	< 0.05		Age	< 0.05

 We then further validated the four-gene signature in an independent dataset GSE202203 from GEO, which is a dataset of primary breast tumors with expression profiling from high throughput sequencing. ​Validation analyses showed that patients with low risk scores had better survival outcomes than those with high risk scores, and that the gene signature yielded good prediction results(ROC: 0.674) (Fig. 8 G-I). 

## Discussion

 The Th1/Th2 balance status of tumor patients has been a concern of researchers and clinicians in recent years. Previous studies have shown that Th1/Th2 unbalance contributes to tumor progression and could be one of the mechanisms that cause immune escape. A shift in Th1/Th2 cell subsets has been reported in lung cancer, glioma, cervical cancer, breast cancer, gastric cancer, colorectal cancer, ovarian cancer, and liver cancer [ 20–22 ]. In the anti-tumor immune response, Th1 cells dominate the cellular immune function of the body and secrete Th1 cytokines, which play a vital role in the anti-tumor immune response. In contrast, Th2 cells work against Th1 cells. The hyposecretion of IL-2 and IFN-γ in peripheral blood is often detected in patients with advanced tumors, and the secretion of IL-10 increases, indicating that Th0 to Th2 differentiation is dominant during tumor growth [ 23 ]. The dominant state of Th2 is closely related to tumor immune escape, but the exact mechanism still needs clarification [ 24 ]. The shift of Th1/Th2 balance and its resulting genomic phenotypic changes may have an impact on tumor development. 

 Basal-like breast cancer belongs to triple-negative breast cancer (TNBC), which is considered to be a highly heterogeneous type of breast cancer. Based on the gene expression profile, Lehmann’s study divided TNBC into six subtypes: basal-like 1 (BL1), basal-like 2 (BL2), immunomodulatory (IM), mesenchymal (M), mesenchymal stem-like (MSL), and luminal androgen receptor (LAR) [ 25 ]. Among these, the IM subtype has a high expression of immune response-related genes. Analogously, the FUSCC subtyping proposed by Jiang and colleagues, which classified TNBC into [ 1 ] luminal androgen receptor (LAR), [ 2 ] immunomodulatory (IM), [ 3 ] basal-like immune-suppressed (BLIS), and [ 4 ] mesenchymal-like (MES), also includes a class of IM subtype with the high expression of PD1, PD-L1, CTLA4, and IDO1, which may benefit from immune-therapy targeting PD1 and/or PD-L1 [ 26 ]. Hence, at least for a significant percentage of basal like breast cancer patients, immunoregulation is strongly associated with their development and outcome. However, many tumor-related immune regulation mechanisms remain to be defined. ​Thus, we hope to further understand the mechanism of immune-related regulation in breast cancer by exploring the shift of the Th1/Th2 balance. 

 In this study, we began with Th1/Th2 balance, an important concept in immune regulation, and identified enriched gene sets and pathways that are related to its regulation. As shown in this study, a suppression mainly in the IFN-α response, IFN-γ response, allograft rejection, IL6 JAK STAT3 signaling, and inflammatory response could influence the Th1/Th2 balance. Furthermore, KEGG analysis demonstrates that the downregulation of IFN-γ and IL2 can be found in almost every pathway enriched. All the evidence that has emerged in this study relates to Th2 polarization [ 6, 27 ]. In addition, it is noted that the downregulation of PD-L1 can be found in the cell adhesion molecule pathway and downregulation of PD-1 can be found in the T cell receptor signaling pathway, and the D-L1 expression and PD-1 checkpoint pathway in the cancer pathway was also downregulated (Supplementary Figs 2 A, 4 A, 5 B). This may indicate that immune-therapy targeting PD1 and/or PD-L1 is not effective in breast cancer with a Th1/Th2 balance toward Th2. 

 We identified CCL1 and MYH6 as independent protective factors based on the different gene expression pattern with high or low Th1 / Th2 ratios, while IFNK and SOAT2 were independent risk factors from univariate and multivariate Cox regression analysis. Among them, CCL1 is a major Treg-attracting chemokine in human invasive breast cancer, positively correlated with Treg infiltration and ER-negative high-grade tumors [ 28 ]. On the contrary, IFNK, MYH6, and SOAT2 have rarely been reported in association with breast cancer. Previous studies showed that IFNK can be regulated by lncRNA and might affect the response to anthracycline treatment in ER-negative breast cancer [ 29 ]. MYH6 and SOAT2 may be associated with the progression of prostate cancer [ 30, 31 ]. Therefore, the expression of the above four genes is associated with the development and prognosis of breast cancer. In addition, the four-gene signature constructed in our study indicates a synergistic prognostic value of the four genes in basal-like breast cancer. 

However, due to the lack of adequate research, their roles in breast cancer still need to be further clarified. We expect that these mystery genes may be novel markers for basal-like breast cancer. Furthermore, our study is based on a comprehensive bioinformatic analysis, further validation is needed to confirm our theory.

## Conclusion

​The Th1 / Th2 ratio is a prognostic factor for breast cancer, and was statistically significant in LumA and Basal-like breast cancer survival analysis. Downregulation of immune-related gene sets and pathways affects the balance of Th1/Th2 towards Th2 polarization and leads to poor outcome.

We further constructed a four-gene signature comprising CCL1, IFNK, MYH6, and SOAT2 genes, which shows a promising predictive value for basal-like breast cancer and may be related to the underlying Th1/Th2 balance regulation mechanism, which is worthy of further study.

 We would like to thank the associate editor and the reviewers for their useful feedback that improved this paper. We thank Guozi Learn Bioinformatics (WeChat Official Accounts). We also thank International Science Editing ( http://www.internationalscienceediting.com ) for editing this manuscript. 

## Supplementary Information

**Additional file 1:** **Table S1.**

**Additional file 2:** **Table S2.**

**Additional file 3:** **Table S3.**

**Additional file 4:** **Table S4.**

**Additional file 5:** **Figure S1.** Cytokine-cytokine receptor pathway. **Figure S2.** Viral protein interaction with cytokine and cytokine receptor pathway. **Figure S3.** Chemokine signaling pathway. **Figure S4.** Cell adhesion molecules pathway. **Figure S5.** Antigen processing and presentation pathway. **Figure S6.** Jak-stat signaling pathway. **Figure S7**. Hematopoietic cell lineage pathway. **Figure S8.** Natural killer cell mediated cytotoxicity pathway. **Figure S9.** Th1 and Th2 cell differentiation pathway. **Figure S10.** Th17 cell differentiation pathway. **Figure S11.** T cell receptor signaling pathway. **Figure S12.** Intestinal immune network for IgA production pathway. **Figure S13.** Type I diabetes mellitus pathway. **Figure S14.** African trypanosomiasis pathway. **Figure S15.** PD-L1 expression and PD-1 checkpoint pathway in cancer. **Figure S16.** Autoimmune thyroid disease pathway. **Figure S17.** Inflammatory bowel disease pathway. **Figure S18.** Rheumatoid arthritis pathway. **Figure S19.** Allograft rejection pathway. **Figure S20.** Graft-versus-host disease pathway. **Figure S21.** Primary immunodeficiency pathway. **Figure S22.** Viral myocarditis pathway. **Figure S23.** Gene sets enriched in GSEA analysis.

## Data Availability

TCGA and GEO belong to public databases. The patients involved in the database have obtained ethical approval. Users can download relevant data for free for research and publish relevant articles. Our study is based on open-source data, so there are no ethical issues and other conflicts of interest. The datasets analyzed for this study can be found as follows: TCGA-BRCA dataset v31.0 [ http://portal.gdc.cancer.gov/] ImmuCellAI dataset [ http://bioinfo.life.hust.edu.cn/ImmuCellAI] Gencode.v22. annotation data [ https://gdc.cancer.gov/] GEO dataset GSE202203 [ https://www.ncbi.nlm.nih.gov/geo/]
