# Supplementary material for: Identification of the prognostic value of Th1/Th2 ratio and a novel prognostic signature in basal-like breast cancer
Source: Hereditas. 2023 Jan 25;160:2. doi: 10.1186/s41065-023-00265-0 (PMC9875389; doi:10.1186/s41065-023-00265-0)

S1. Cytokine-cytokine receptor interaction pathway

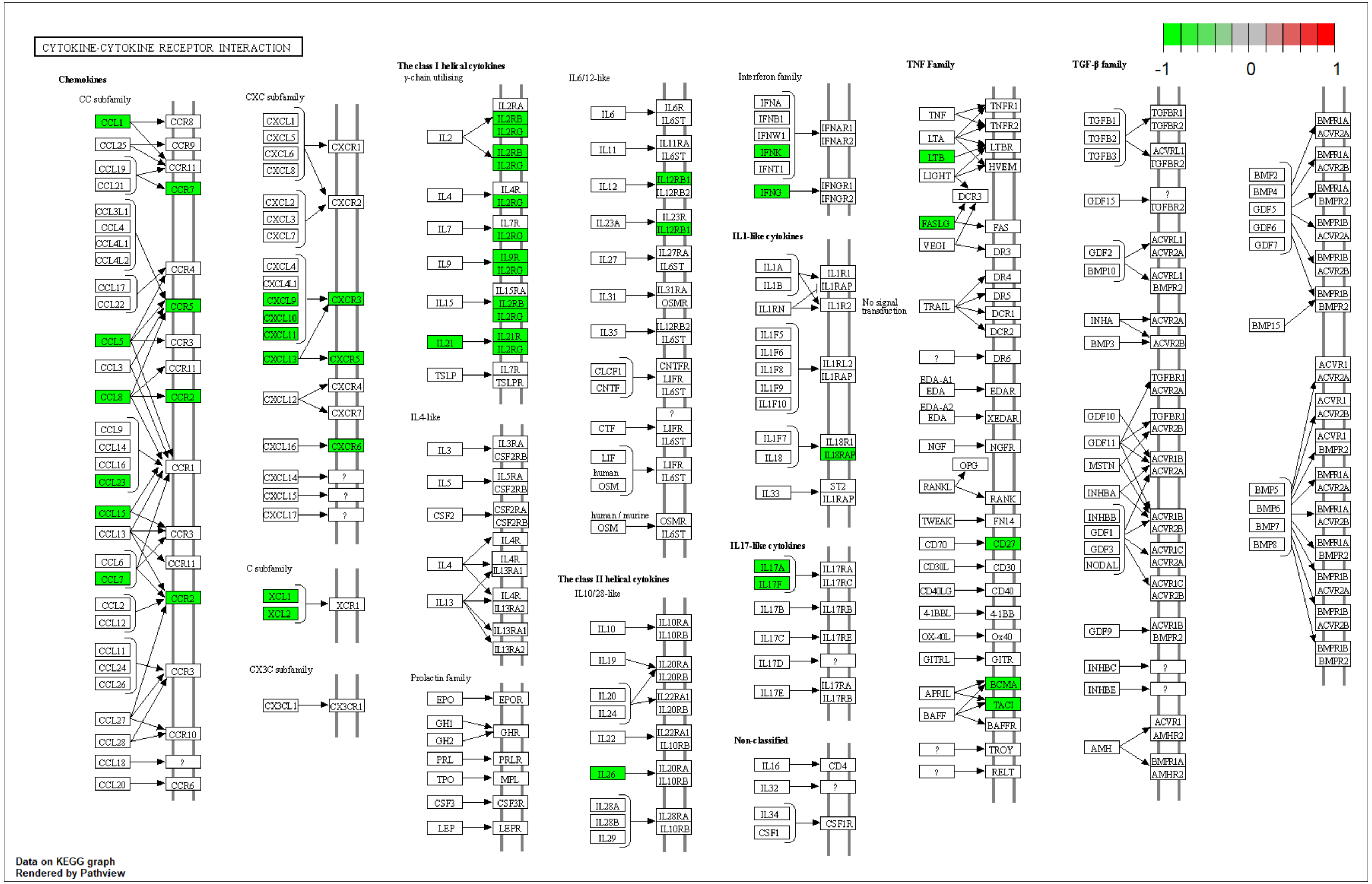

S2. Viral protein interaction with cytokine and cytokine receptor pathway

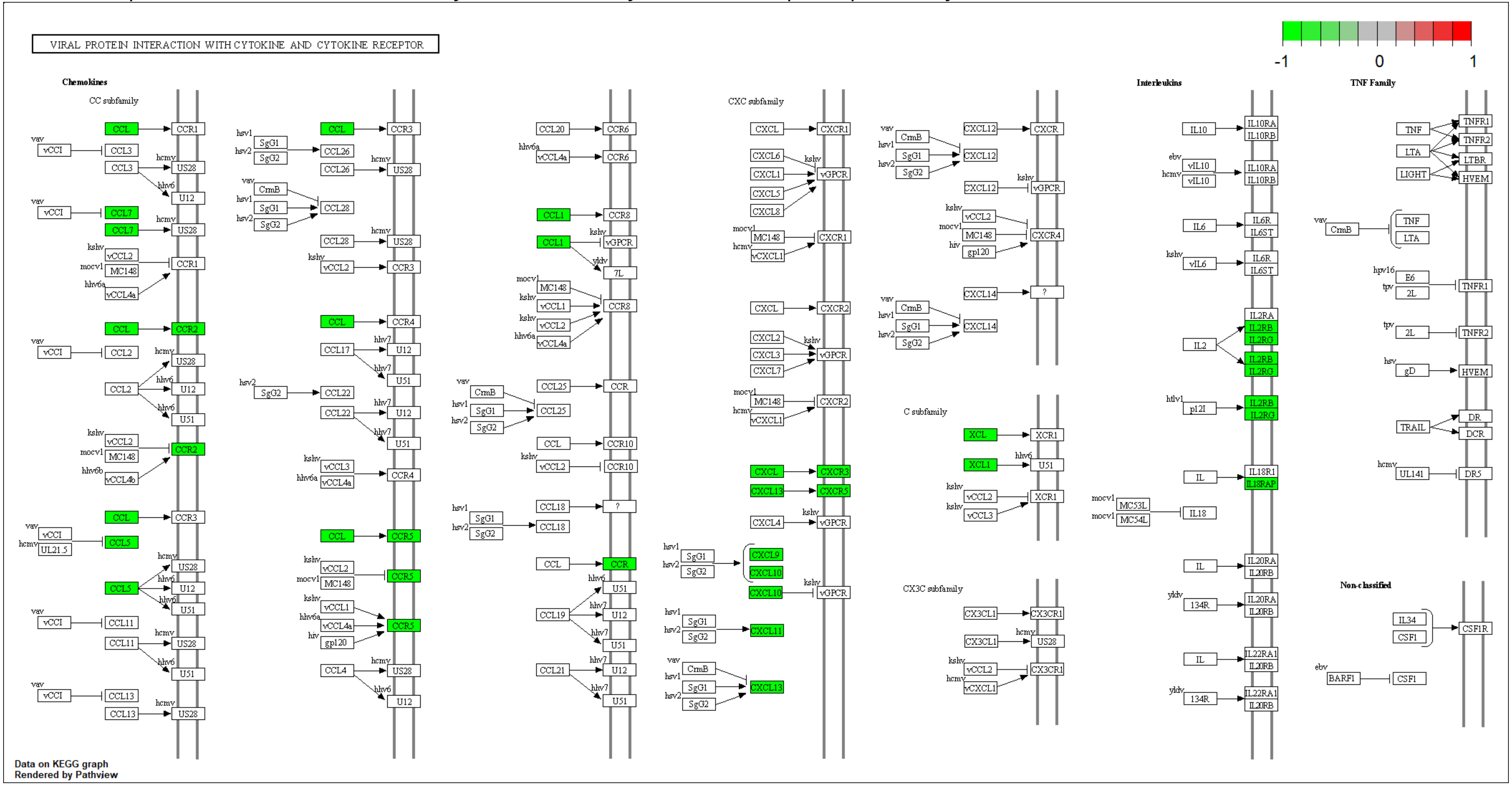

S3. Chemokine signaling pathway

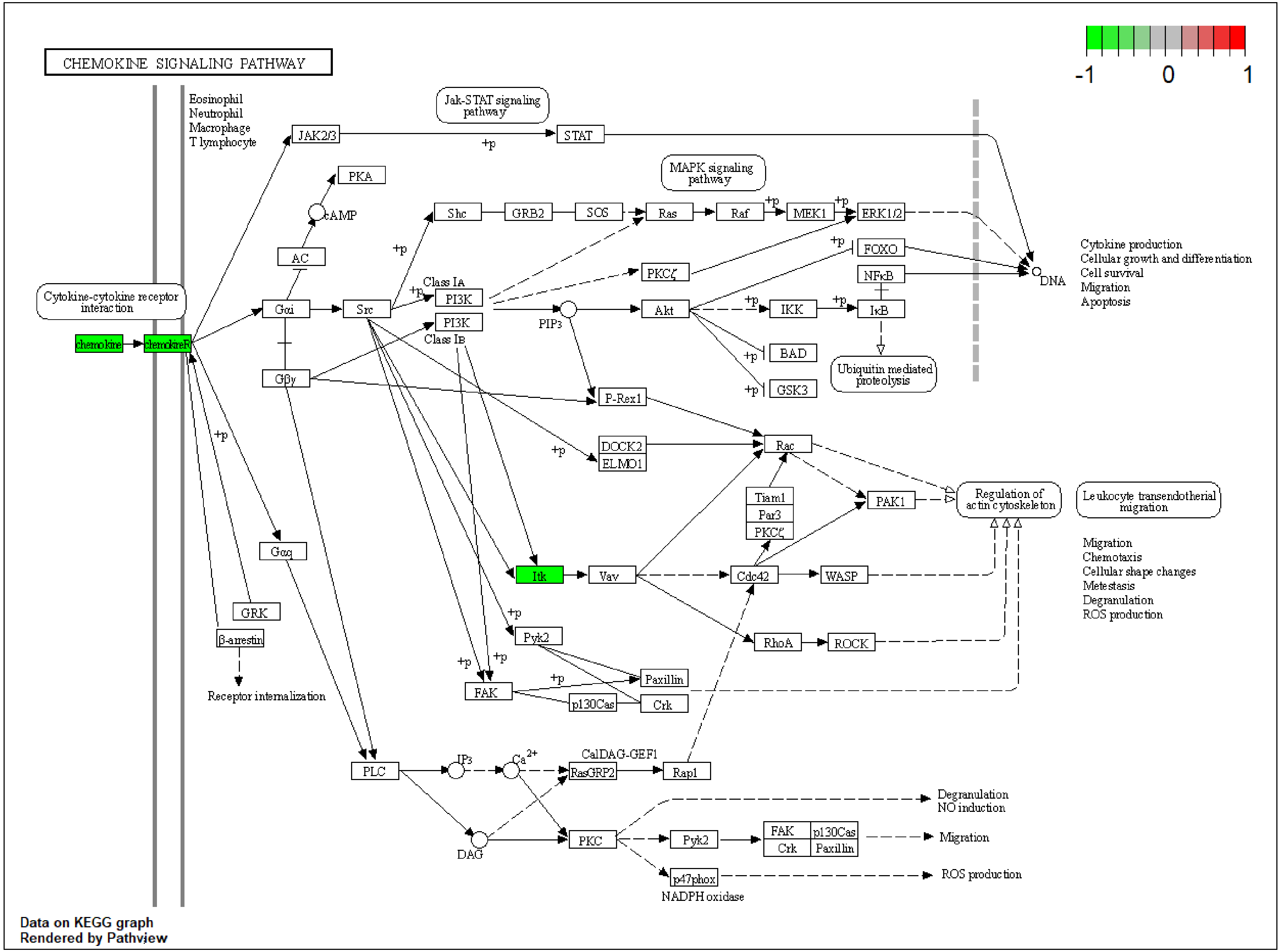

S4. Cell adhesion molecules pathway

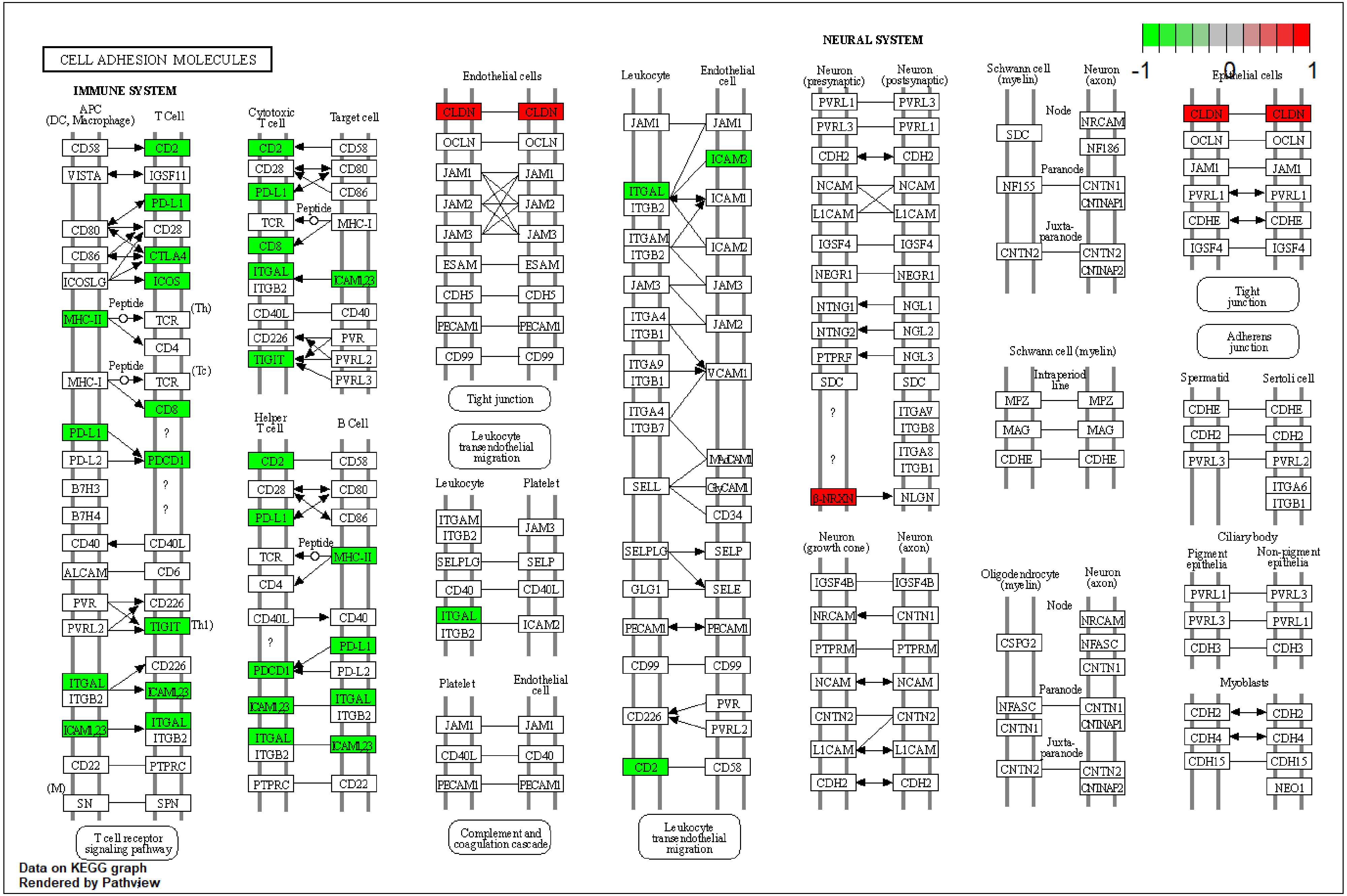

S5. Antigen processing and presentation pathway

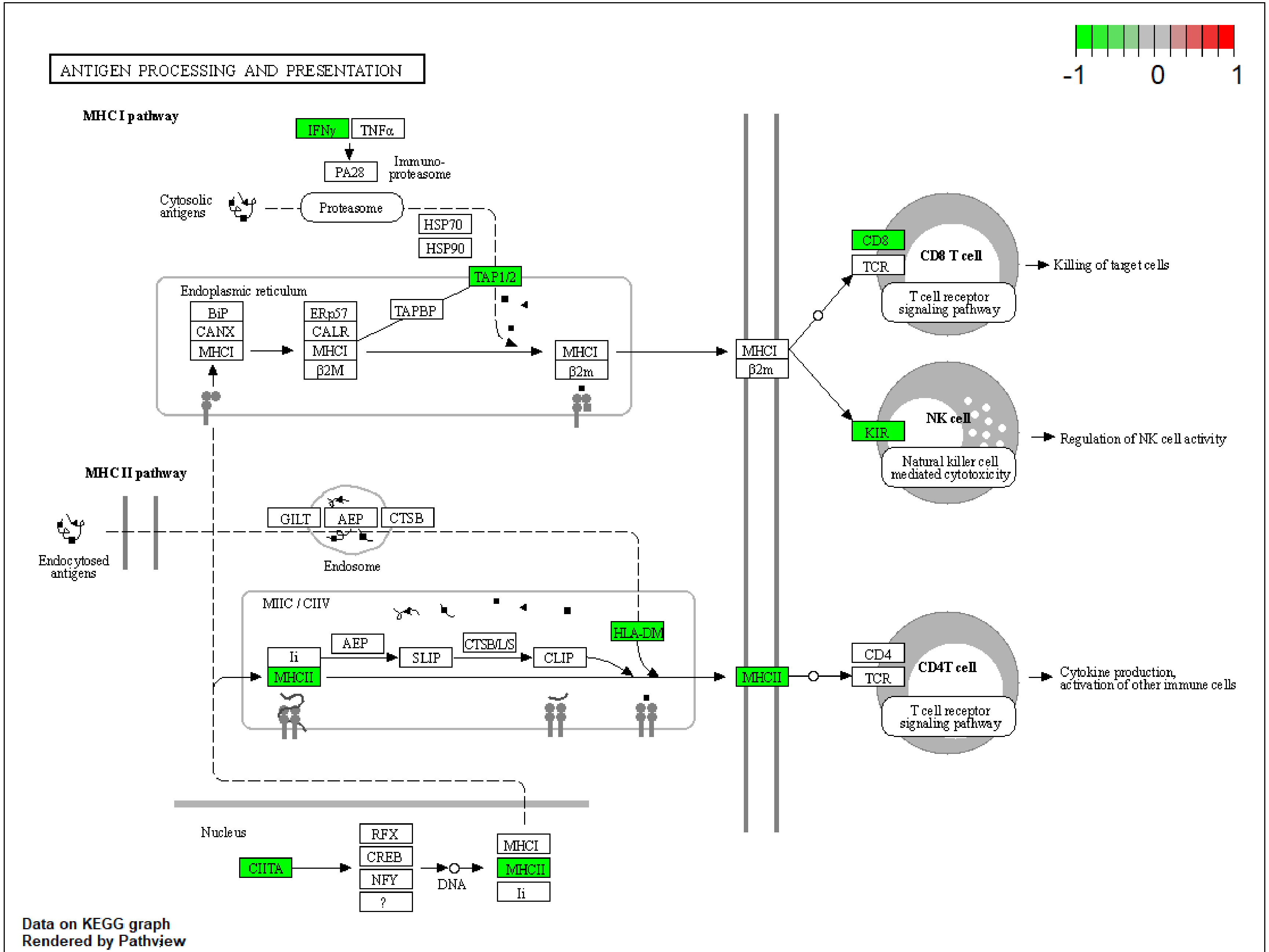

S6. Jak-stat signaling pathway

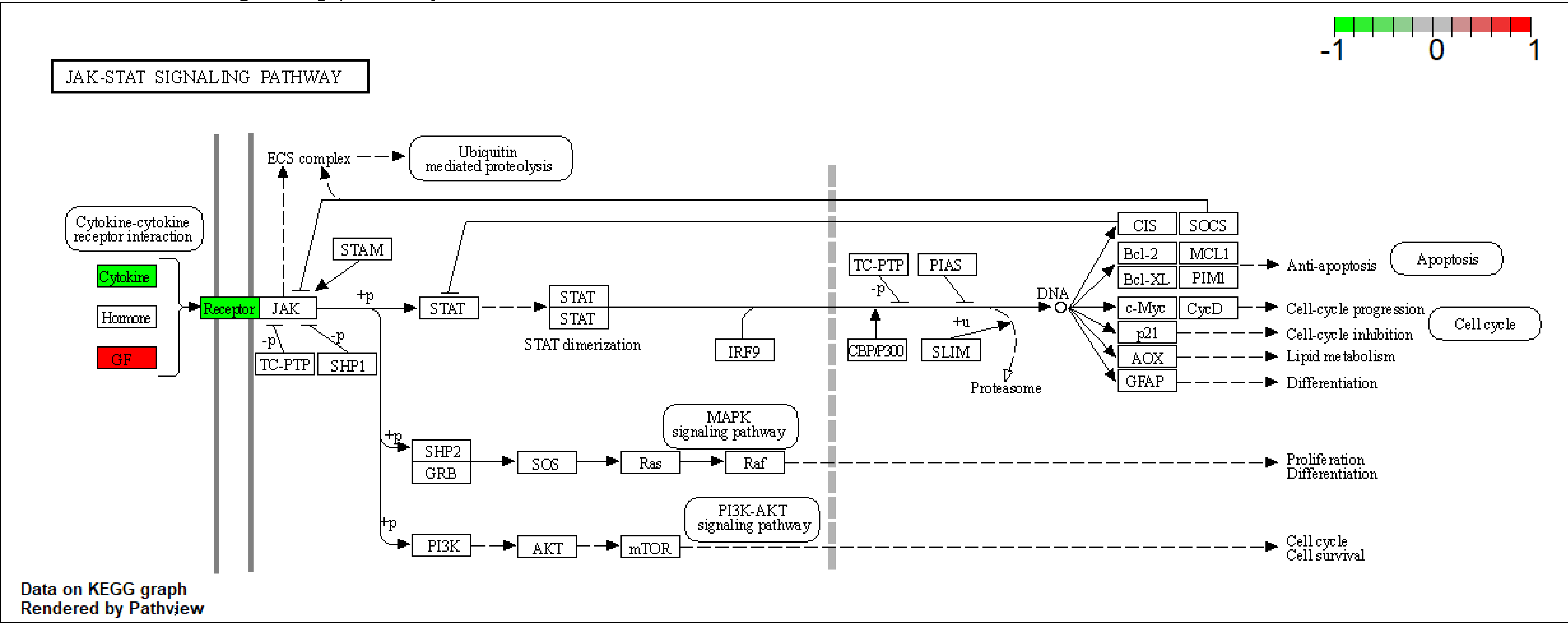

S7. Hematopoietic cell lineage pathway

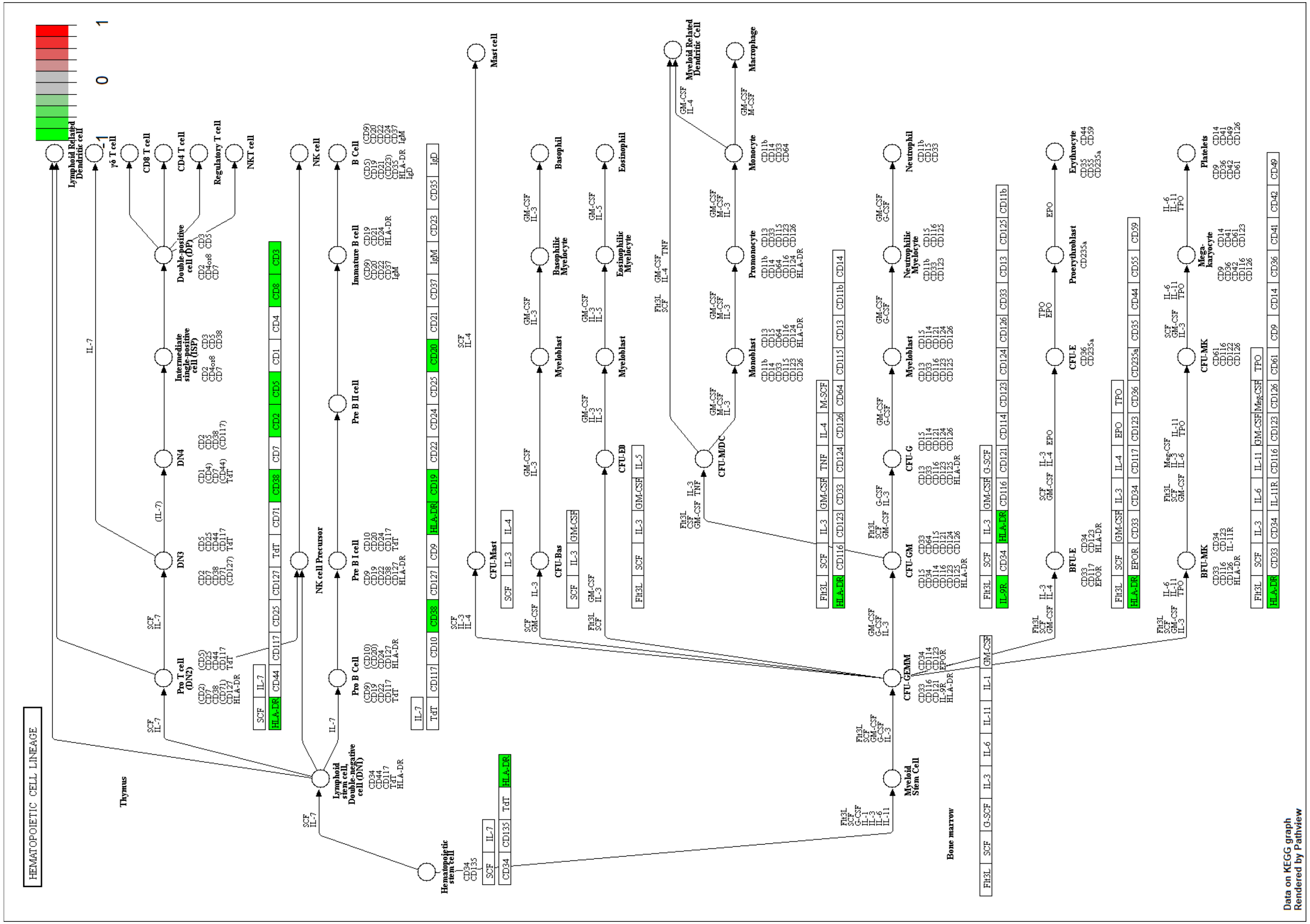

S8.Natural killer cell mediated cytotoxicity pathway

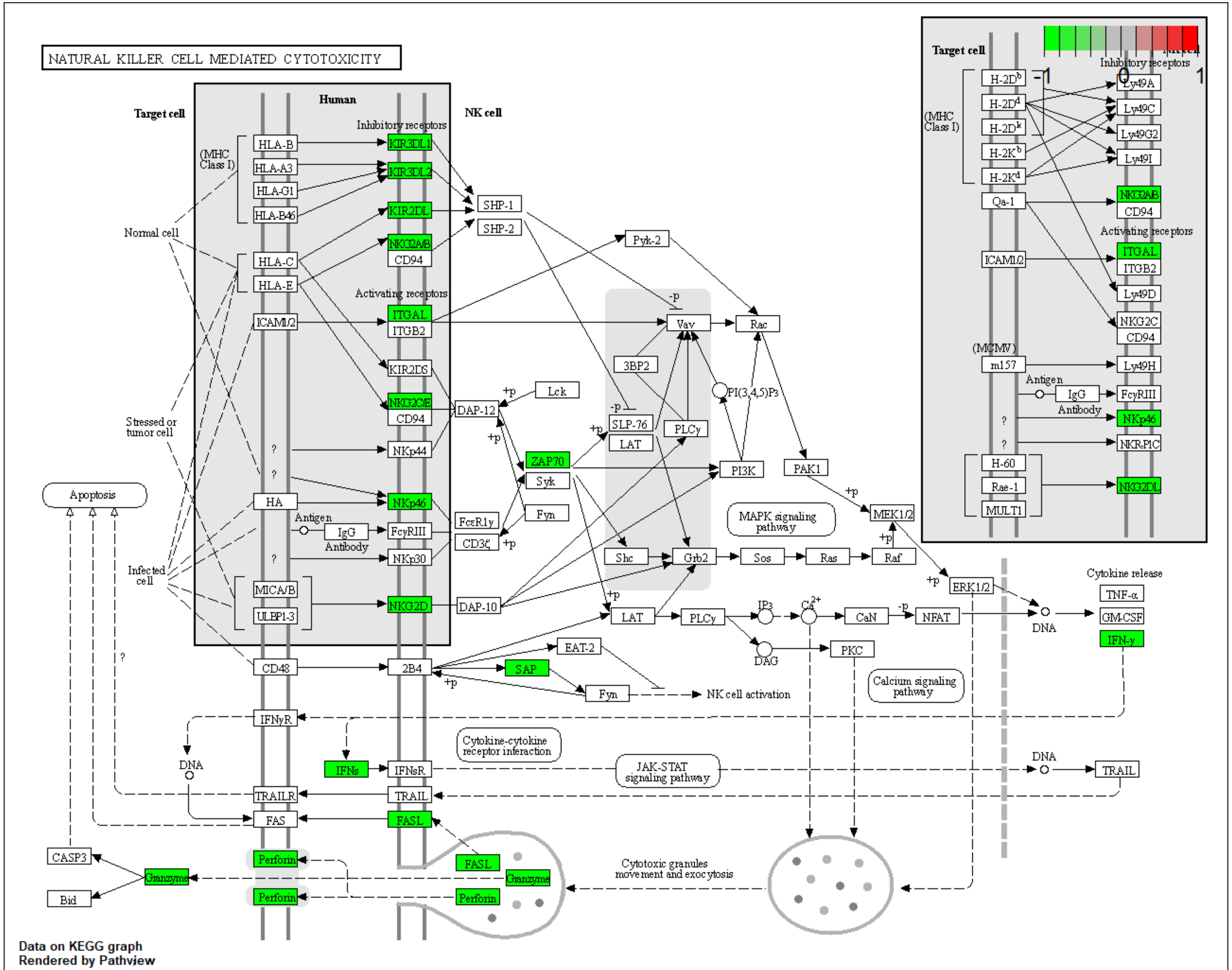

S9. Th1 and Th2 cell differentiation pathway

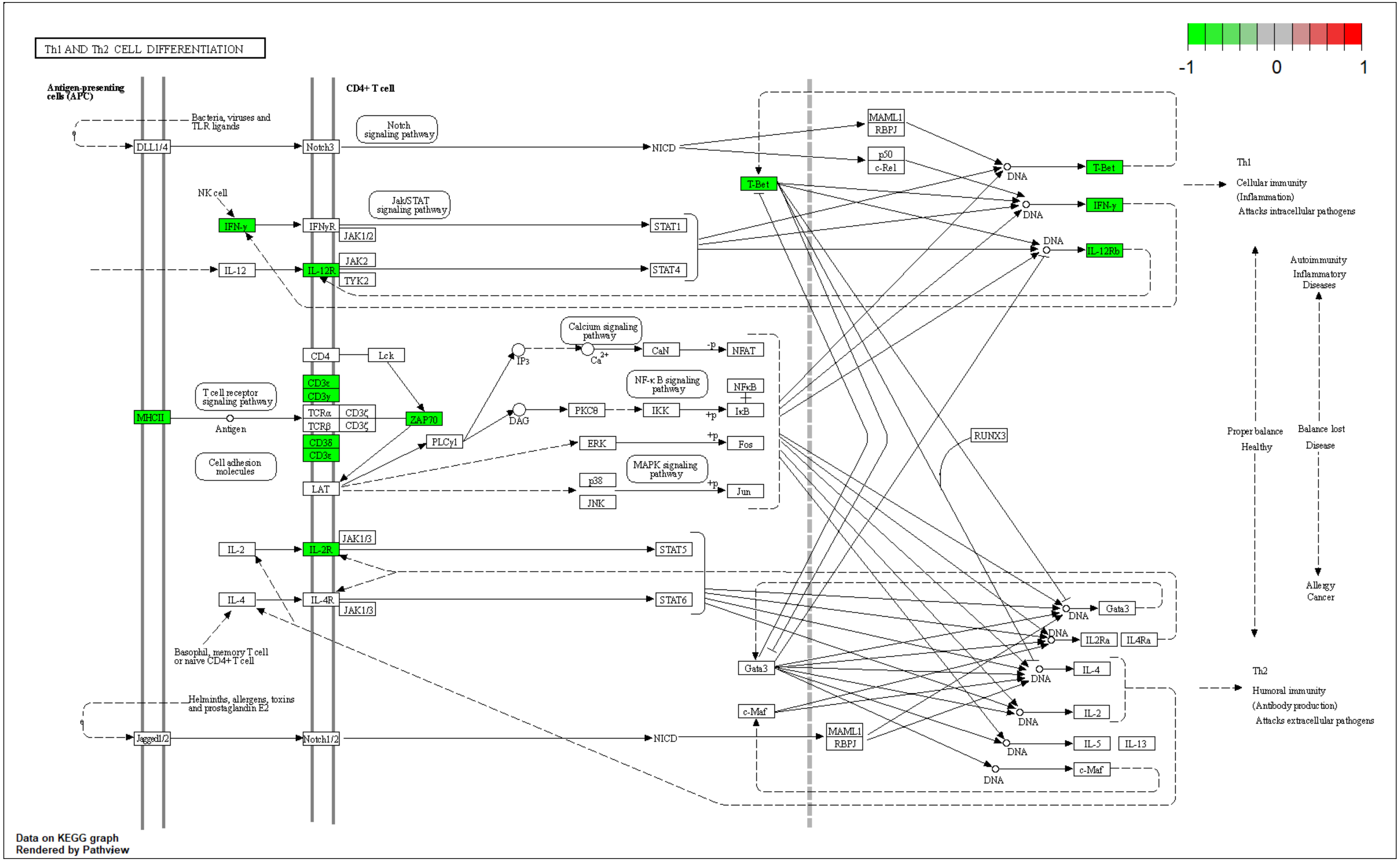

S10. Th17 cell differentiation pathway

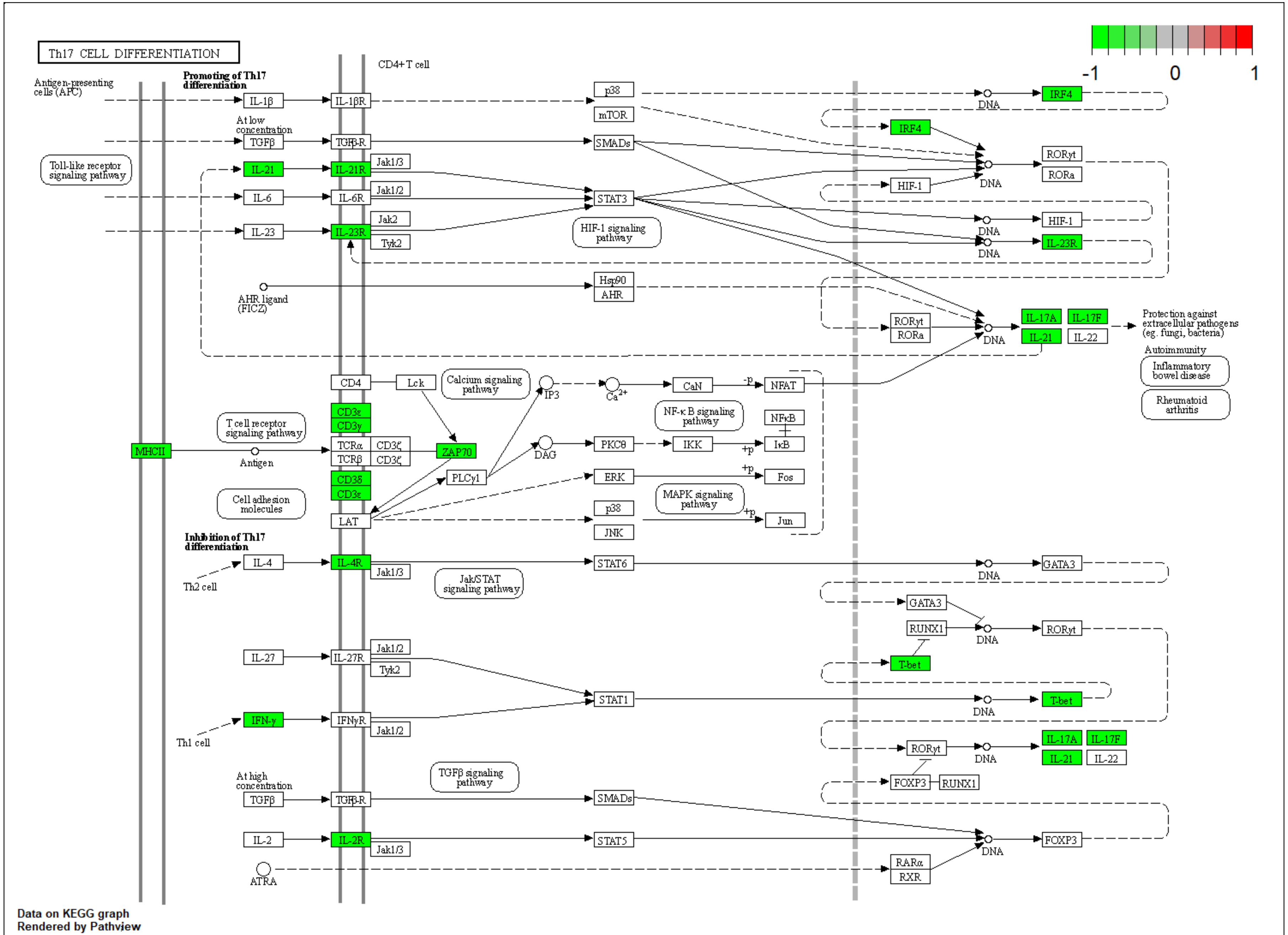

S11. T cell receptor signaling pathway

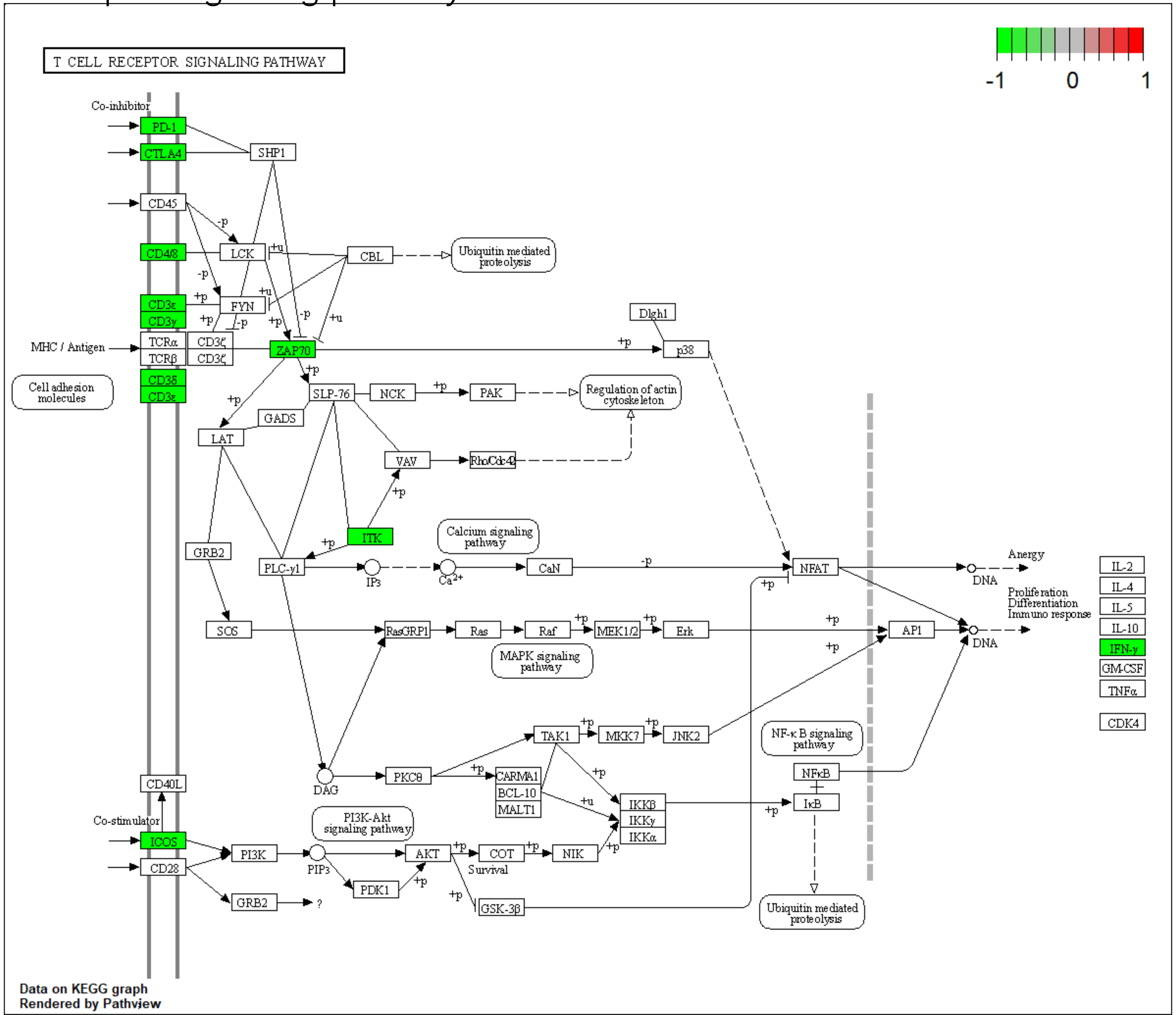

S12. Intestinal immune network for IgA production pathway

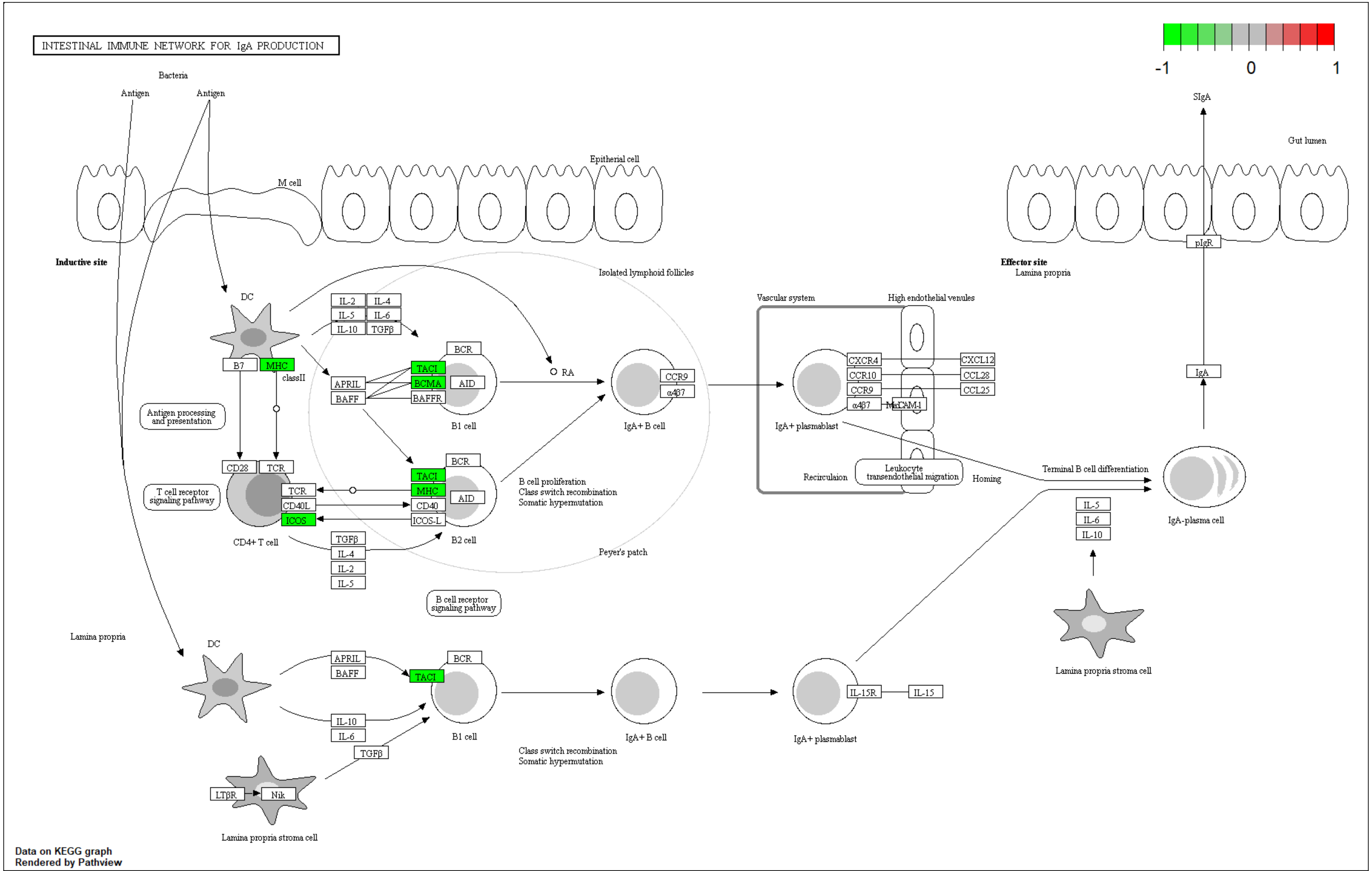

S13. Type I diabetes mellitus pathway

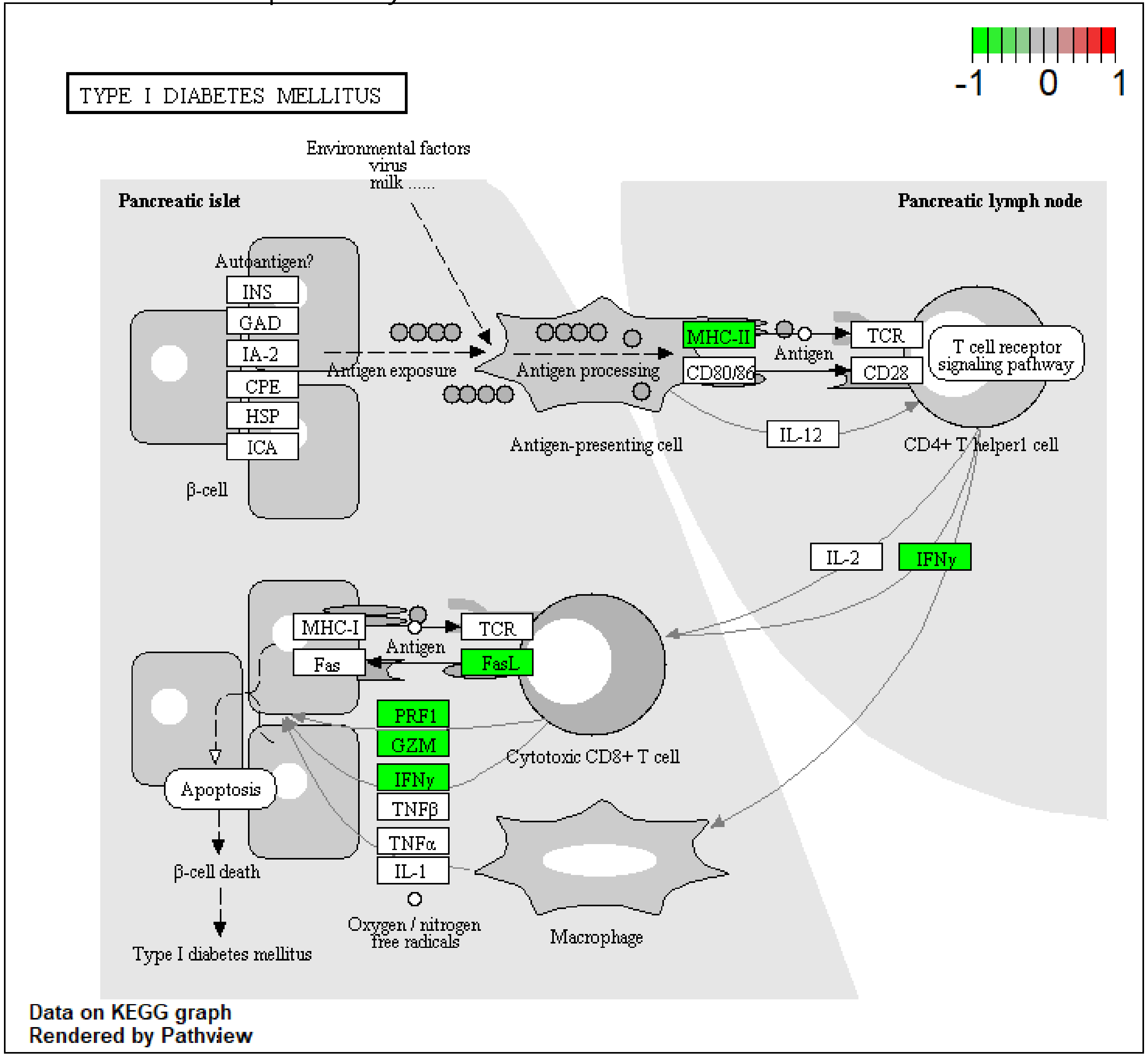

S14. African trypanosomiasis pathway

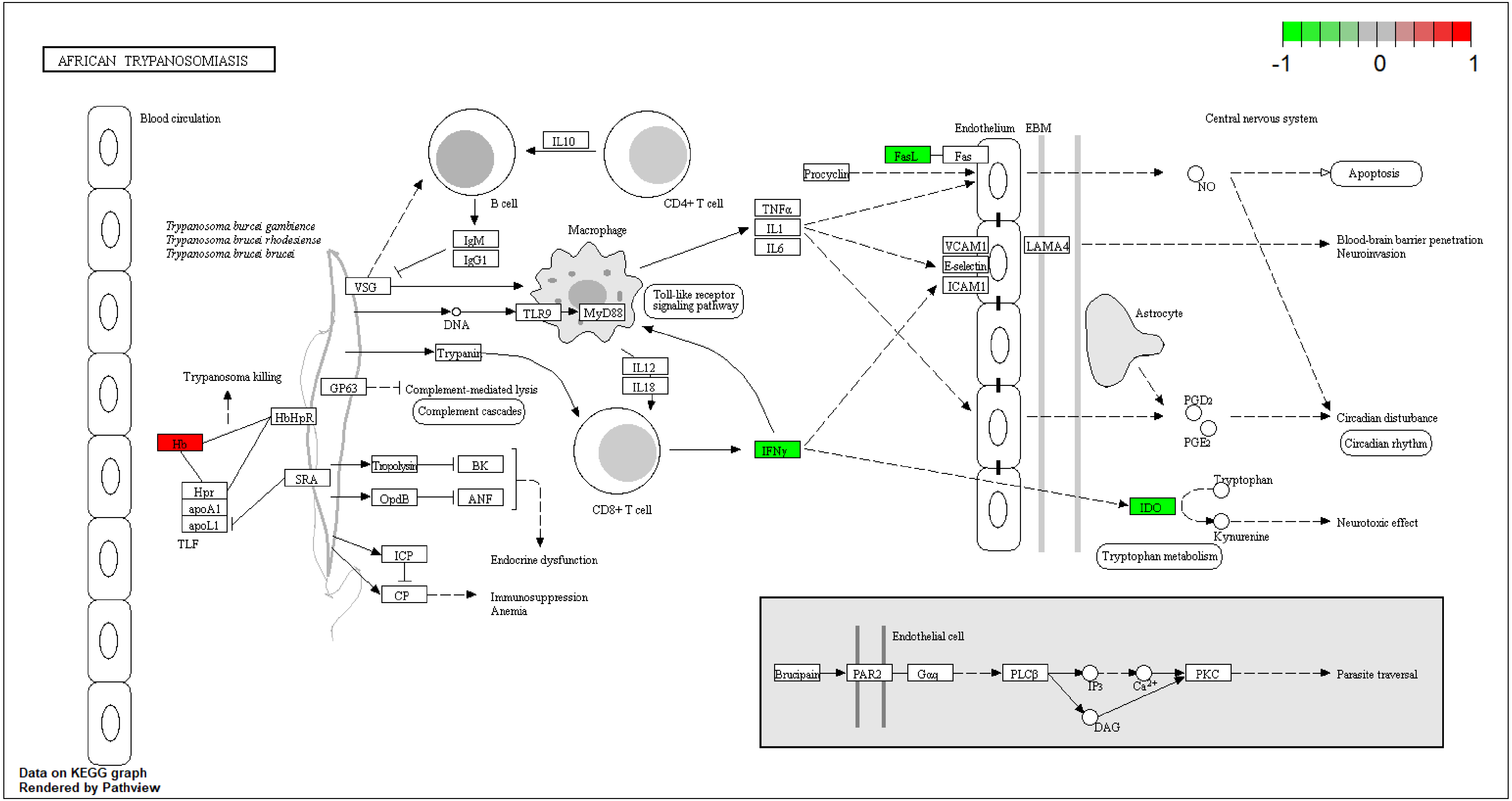

S15. PD-L1 expression and PD-1 checkpoint pathway in cancer

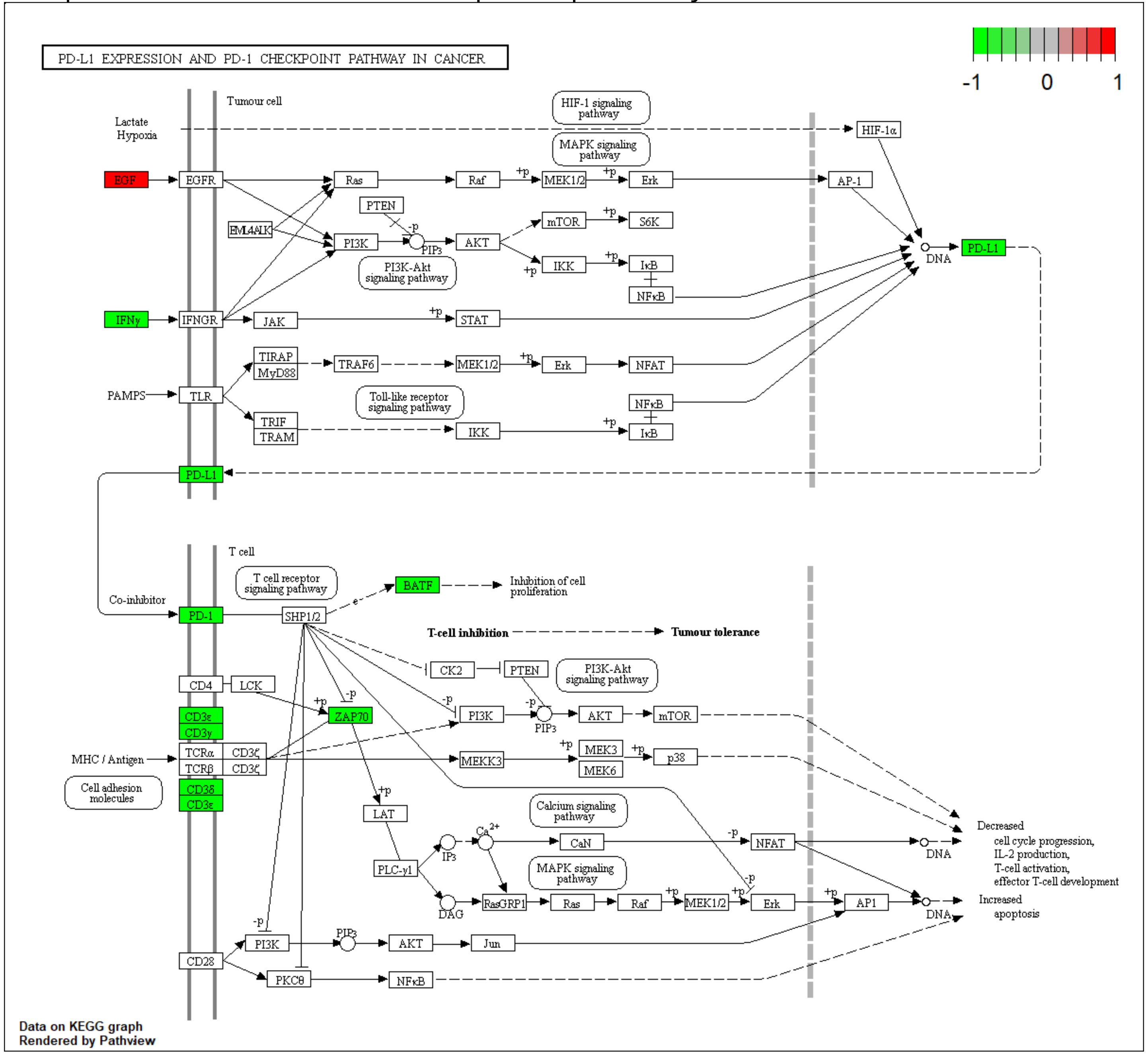

S16. Autoimmune thyroid disease pathway

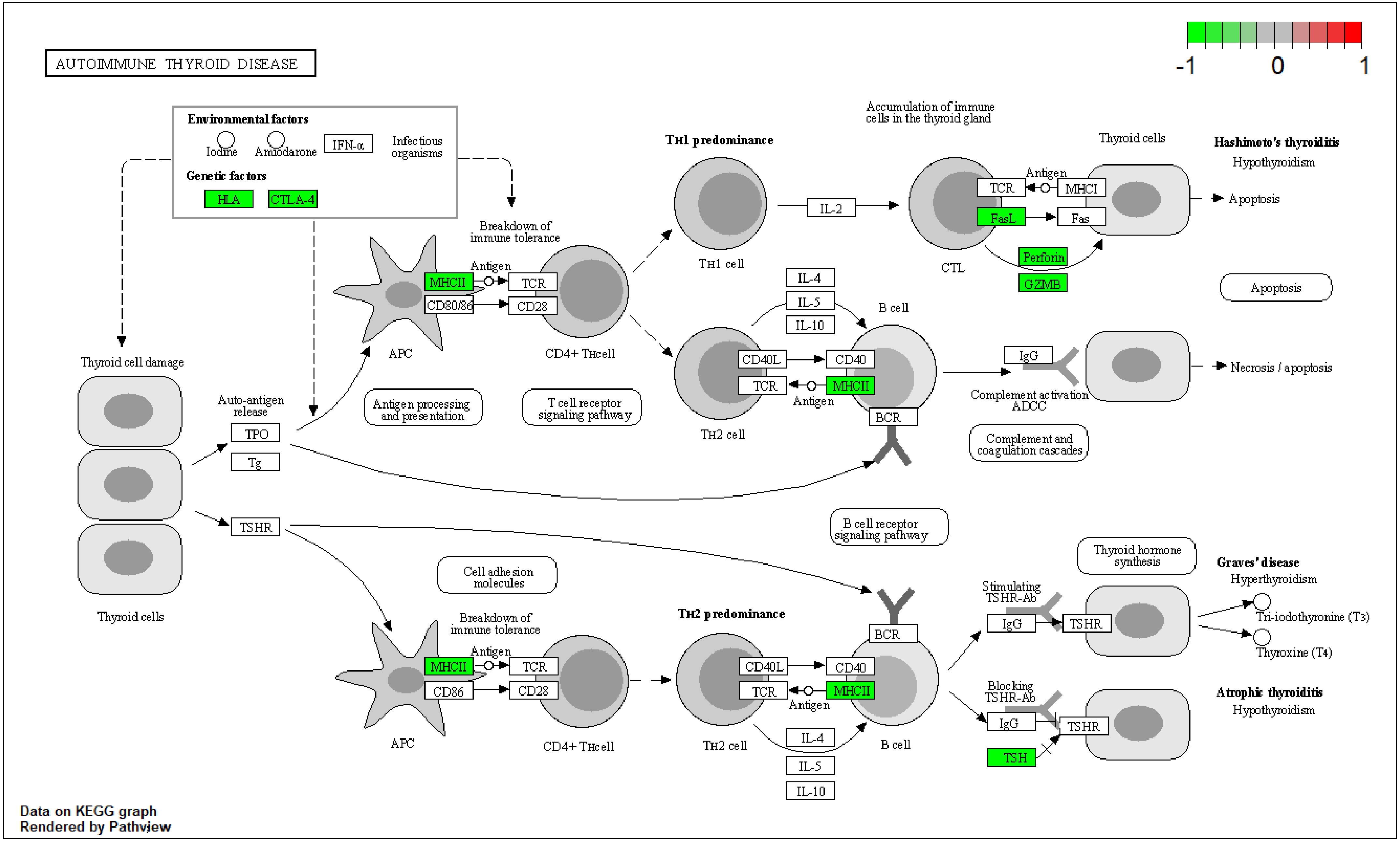

S17. Inflammatory bowel disease pathway

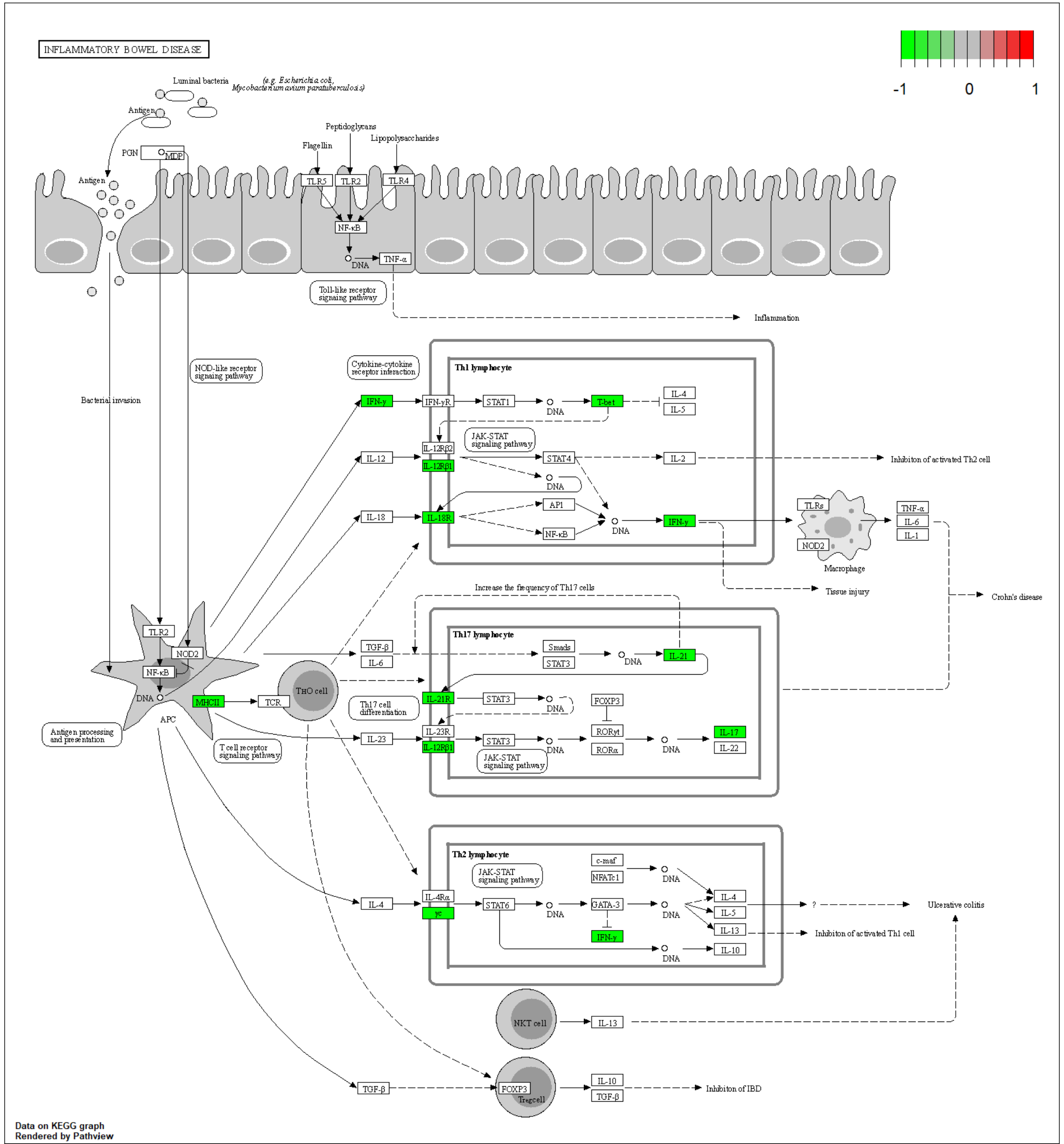

S18. Rheumatoid arthritis pathway

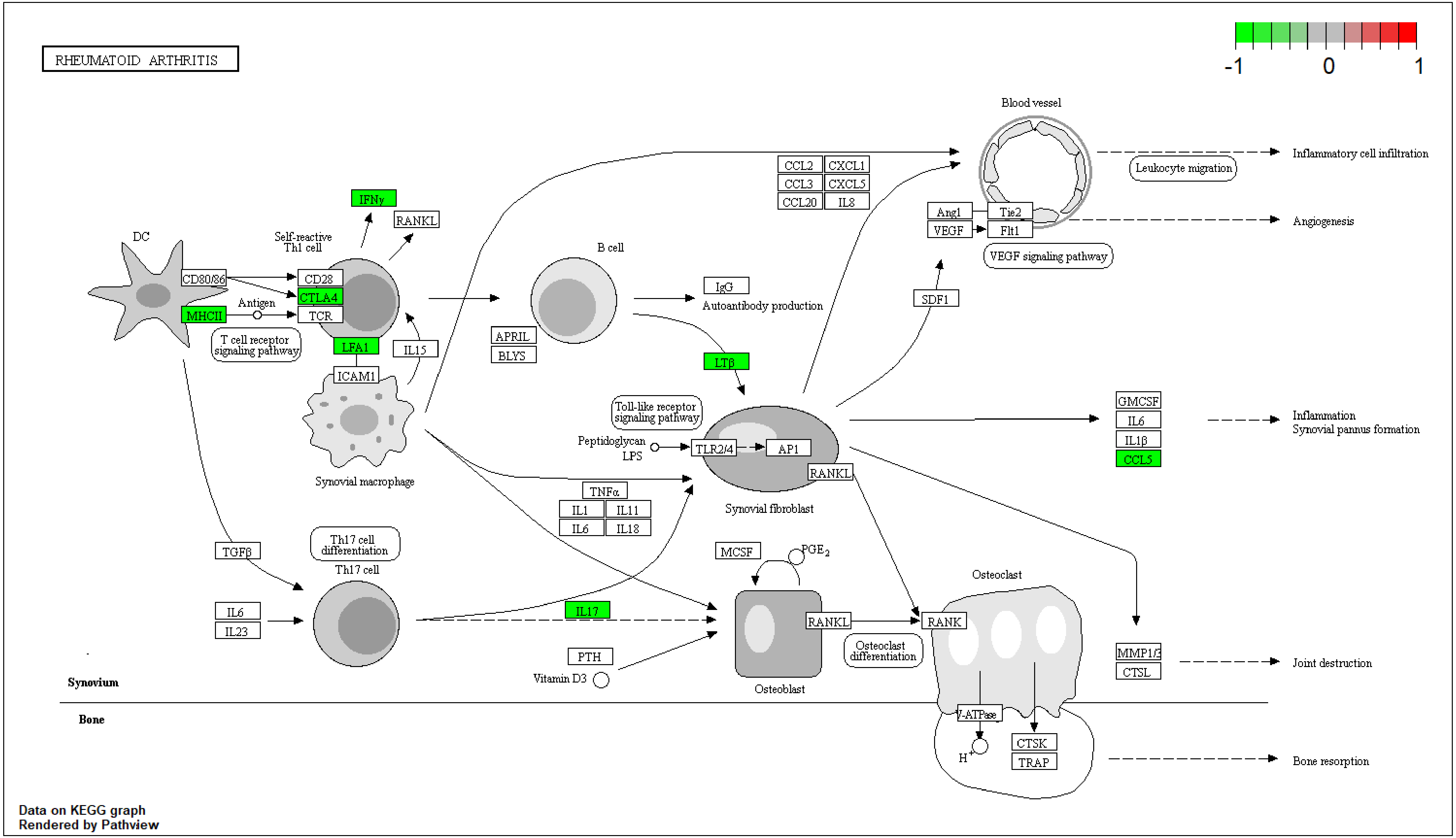

S19. Allograft rejection pathway

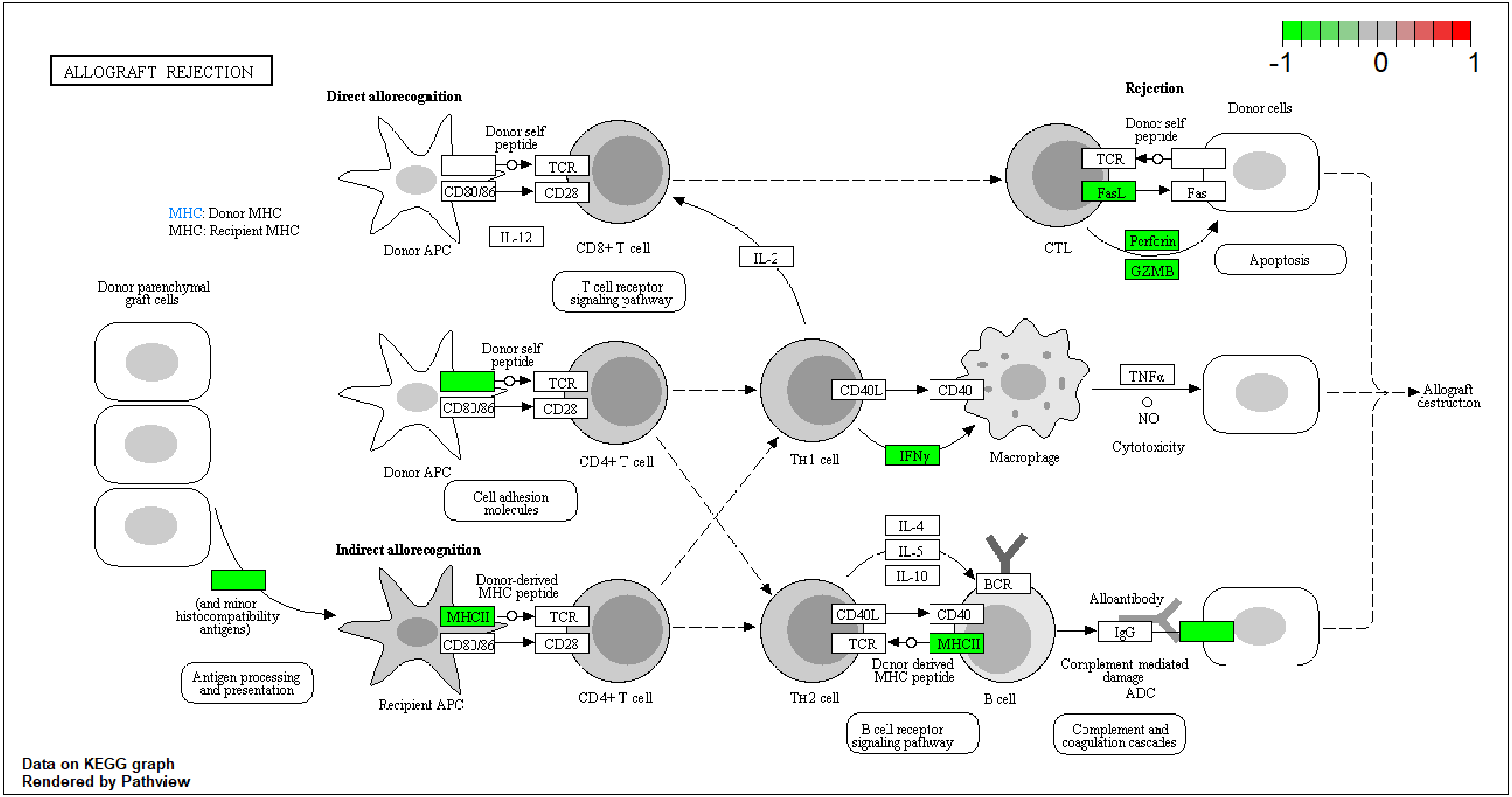

S20. Graft-versus-host disease pathway

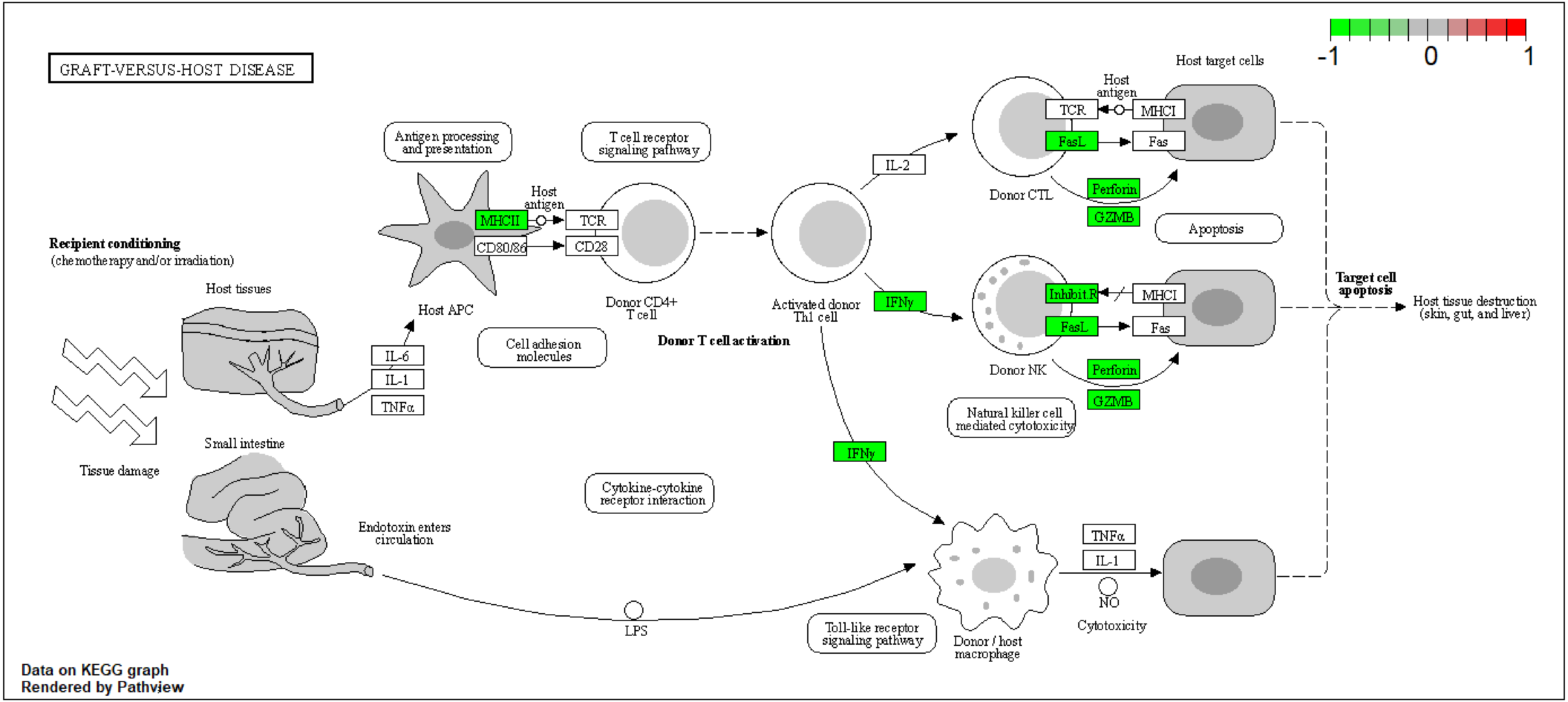

S21. Primary immunodeficiency pathway

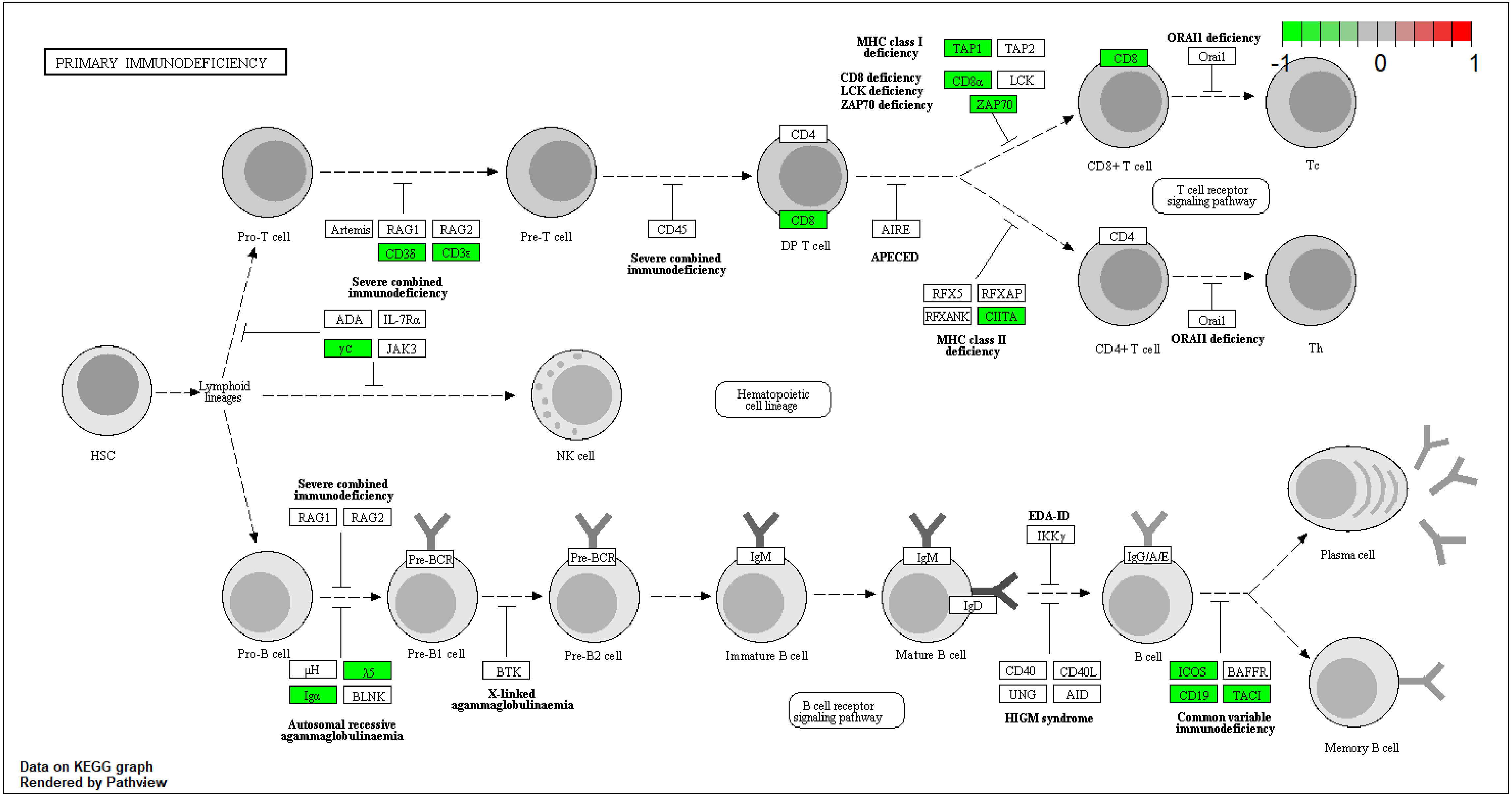

S22. Viral myocarditis pathway

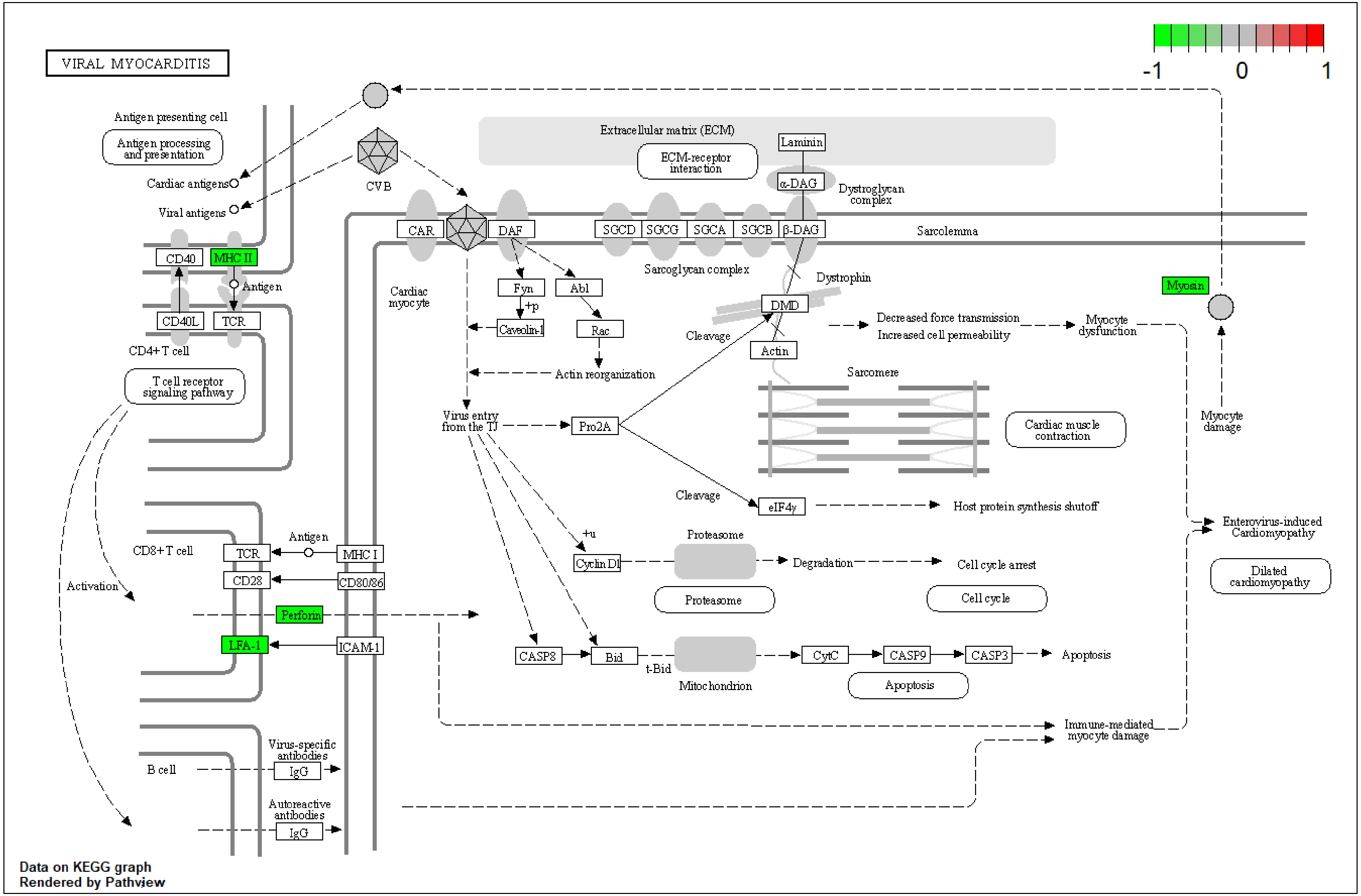

S23. Gene sets enriched in GSEA analysis

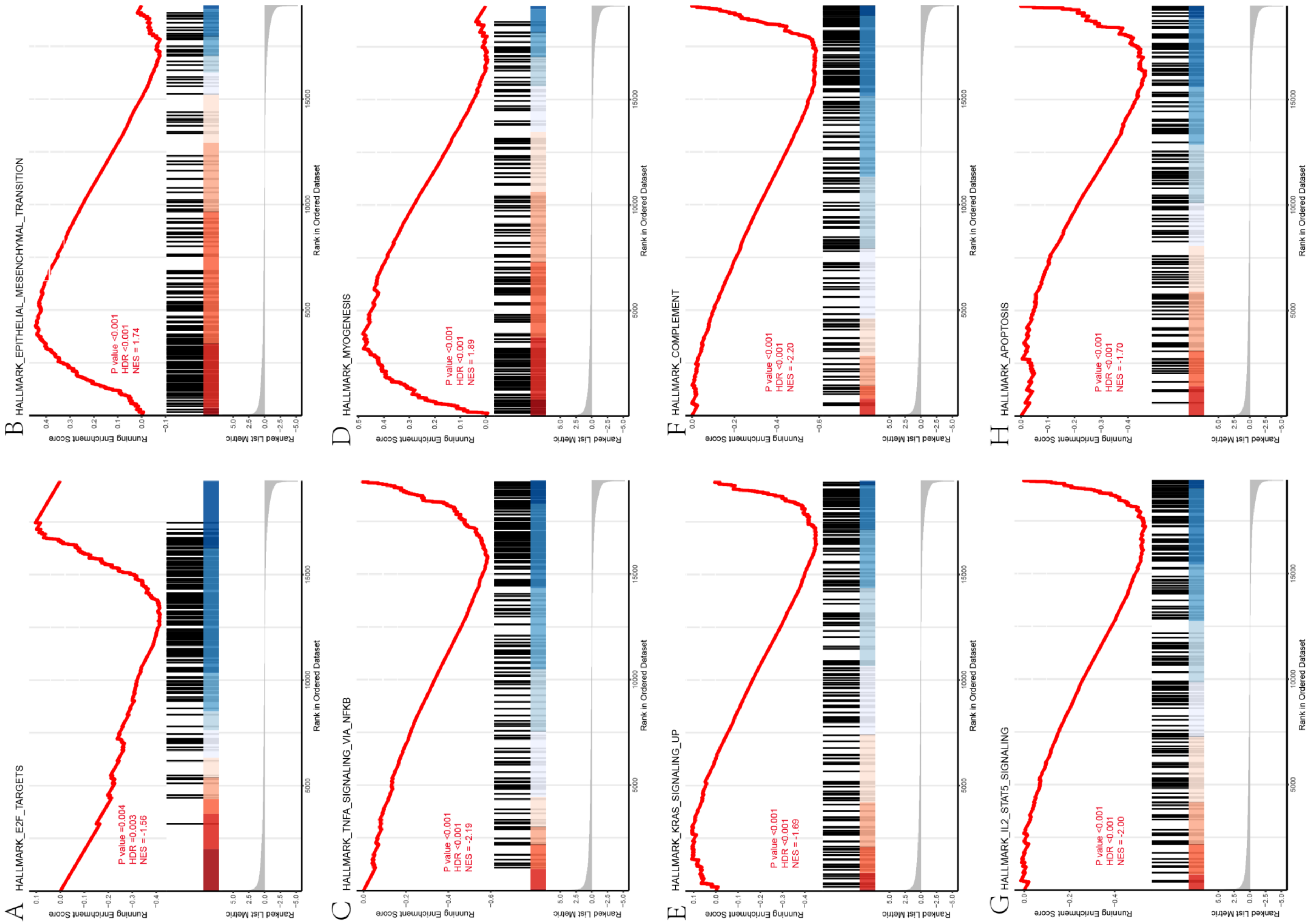

Supplement: Supplementary file 5 — Additional file 5: Figure S1. Cytokine-cytokine receptor pathway. Figure S2. Viral protein interaction with cytokine and cytokine receptor pathway. Figure S3. Chemokine signaling pathway. Figure S4. Cell adhesion molecules pathway. Figure S5. Antigen processing and presentation pathway. Figure S6. Jak-stat signaling pathway. Figure S7. Hematopoietic cell lineage pathway. Figure S8. Natural killer cell mediated cytotoxicity pathway. Figure S9. Th1 and Th2 cell differentiation pathway. Figure S10. Th17 cell differentiation pathway. Figure S11. T cell receptor signaling pathway. Figure S12. Intestinal immune network for IgA production pathway. Figure S13. Type I diabetes mellitus pathway. Figure S14. African trypanosomiasis pathway. Figure S15. PD-L1 expression and PD-1 checkpoint pathway in cancer. Figure S16. Autoimmune thyroid disease pathway. Figure S17. Inflammatory bowel disease pathway. Figure S18. Rheumatoid arthritis pathway. Figure S19. Allograft rejection pathway. Figure S20. Graft-versus-host disease pathway. Figure S21. Primary immunodeficiency pathway. Figure S22. Viral myocarditis pathway. Figure S23. Gene sets enriched in GSEA analysis. [file 41065_2023_265_MOESM5_ESM.pdf]
